# Sustained hypoxia induces divergent response patterns in cardiac metabolic-immune adaptation and circadian rhythm regulation

**DOI:** 10.1371/journal.pone.0357966

**Published:** 2026-09-11

**Authors:** Shuting Cheng, Xin Zhuo, Yuhan Deng, Fan Hu, Hanmin Liu, Lihong Wan, Zhou Jiang

**Affiliations:** 1 Department of Biomedical Engineering, West China School of Basic Medical Sciences & Forensic Medicine, Sichuan University, Chengdu, PR China; 2 NHC Key Laboratory of Chronobiology, Sichuan University, Chengdu, Sichuan, PR China; 3 West China Second University Hospital, Sichuan University, Chengdu, Sichuan, PR China; 4 Department of Pediatrics, West China Second University Hospital, Sichuan University, Chengdu, Sichuan, PR China; 5 Key Laboratory of Birth Defect and Related Diseases of Women and Children (Sichuan University), Ministry of Education, Chengdu, Sichuan, PR China; 6 Department of Pharmacology, West China School of Basic Medical Sciences & Forensic Medicine, Sichuan University, Chengdu, Sichuan, PR China; Indiana University School of Medicine, UNITED STATES OF AMERICA

## Abstract

**Objective:**

Chronic hypoxia is a critical pathological factor in cardiovascular disease, yet the molecular mechanisms underlying cardiac adaptation to sustained hypoxic stress remain incompletely understood.

**Methods:**

With 3 GEO datasets, we performed comparative transcriptomic analysis across three hypoxia paradigms (sustained, prenatal, and adult subacute hypoxia) using DESeq2, Pearson correlation analysis, and KEGG pathway enrichment. Permutation testing (n = 10,000) validated robustness. Disease enrichment analysis and cardiovascular drug target analysis were conducted using KEGG pathway-disease mappings and four drug-gene interaction databases.

**Results:**

Sustained hypoxia induced 181 DEGs, prenatal hypoxia produced only 2, and adult subacute hypoxia yielded 67, with no overlap among groups. Eight circadian DEGs were identified in the sustained hypoxia group and 7 distinct circadian DEGs in the prenatal adult programmed heart group, with no overlap between the two sets. The circadian interactome showed dramatic remodeling: gene pairs decreased by 67.5% (160–52), with three adaptive patterns (enhanced synchronicity, interaction reversal, weakened synchronicity). ASS1 emerged as the predominant co-expression hub under hypoxia (88.5% vs 11.25% in normoxia), while Bmal1-Npas2 maintained stable correlation (r = 0.838 vs 0.829). Pathway analysis revealed divergent enrichment profiles: 55 KEGG pathways enriched in metabolic-immune system, only 3 pathways for circadian genes, with no overlap. Disease and drug target analyses further corroborated these divergent profiles.

**Conclusion:**

This study identifies ASS1 as a transcript-level co-expression hub within the circadian interaction network under hypoxia, demonstrates the divergent enrichment profiles of metabolic-immune versus circadian systems at the pathway, disease, and drug target levels, and offers potential therapeutic targets for chronic hypoxia-associated cardiovascular diseases.

## 1. Introduction

The development and progression of cardiovascular diseases are closely linked to the metabolic reprogramming of immune cells. A core tenet of immunometabolism is that the functional state of an immune cell is intrinsically tied to its metabolic program: pro-inflammatory cells predominantly rely on aerobic glycolysis, whereas anti-inflammatory cells depend on oxidative phosphorylation and fatty acid oxidation [1,2]. Within the context of cardiovascular disease, the hypoxic microenvironment of atherosclerotic plaques induces a metabolic shift in macrophages from oxidative phosphorylation towards glycolysis, disrupting the tricarboxylic acid (TCA) cycle and leading to persistent epigenetic modifications [1]. Following myocardial infarction, damage-associated molecular patterns released by necrotic cardiomyocytes trigger HIF-1α-mediated metabolic reprogramming in various immune cells towards glycolysis [1,3].

Additionally, a cell-autonomous circadian clock functions as a core mechanism for metabolic regulation within the heart. Cardiac metabolism exhibits significant time-dependent variations, glucose oxidation is approximately 2.5 times higher during the active phase than during the sleep phase, and triglyceride turnover peaks towards the end of the active phase [4,5]. Epidemiological studies indicate that myocardial infarction and ventricular arrhythmias are more prevalent in the morning, a pattern closely associated with circadian regulation [6,7]. Interventions such as time-restricted feeding can restore metabolic circadian rhythms and improve cardiac function, providing critical theoretical support for chronotherapeutic strategies in cardiovascular disease [4,6].

Under hypoxic conditions, metabolic, immune, and rhythmic regulation form an interconnected network. While HIF-1α is rapidly degraded under normoxia, it is stabilized under hypoxic conditions, where it primarily promotes glycolytic metabolism, reduces oxygen consumption, and decreases reactive oxygen species production, thereby driving a shift from oxidative phosphorylation to anaerobic glycolysis [8]. Following myocardial infarction, ischemia-induced hypoxia stabilizes HIF-1α. Acting as a core transcription factor, HIF-1α directly activates the expression of genes encoding glucose transporters and glycolytic enzymes, while simultaneously inhibiting pyruvate entry into the TCA cycle via activation of pyruvate dehydrogenase kinase 1 [9]. This process results in a disruption in the TCA cycle, leading to the accumulation of key intermediate metabolites such as succinate, citrate, and fumarate. When oxidized by succinate dehydrogenase upon reperfusion, succinate accumulation generates substantial mitochondrial reactive oxygen species, further stabilizing HIF-1α and activating inflammatory signaling; citrate is converted to acetyl-CoA by cytosolic ATP citrate synthase in the cytosol, facilitating the epigenetic expression of inflammatory genes; and fumarate is involved in the epigenetic reprogramming of monocytes and promotes the production of cytokines like TNF-α and IL-6 [9]. Hypoxia, a common feature of diverse pathological environments including the tumor microenvironment and atherosclerotic plaques, drives immunometabolic reprogramming via HIF-1α stabilization. HIF-1α not only enhances the expression of glycolytic enzymes but also promotes the conversion of pyruvate to lactate by lactate dehydrogenase and upregulates glucose transporter expression, thereby increasing glucose uptake and lactate secretion [10,11].

Direct cross-regulation exists between the circadian rhythm and hypoxia signaling pathways. BMAL1 can bind to HIF-1α and enhance its stability, while HIF-1α can bind to the promoters of *Per2* and *Cry1* genes, forming a regulatory circuit [12]. Transcripts of hypoxia-responsive genes in the heart peak in the morning, coinciding with the peak incidence time of human cardiovascular events. This suggests that the expression of circadian-regulated hypoxia-response genes may determine the temporal window of tissue vulnerability to ischemic injury [12].

However, despite hypoxia's known independent regulatory effects on metabolism, circadian rhythms, and immunity, the interactive mechanisms governing cardiac immunometabolic adaptation and circadian regulation under chronic hypoxia have yet to be systematically elucidated. Current research has confirmed that hypoxia triggers cardiac metabolic remodeling via the HIF signaling pathway, induces macrophage phenotypic switching within the heart, and concurrently disrupts core circadian gene expression [8–12]. Furthermore, the interaction network between metabolism and immunity in the normoxic heart has been preliminarily characterized [1–3,13]. Nevertheless, it remains unclear whether a similar regulatory logic applies to the tripartite coupling of metabolism, immunity, and rhythm in the hypoxic heart, or if unique response patterns exist. Whether these systems exhibit divergent response patterns under sustained hypoxia also lacks systematic investigation. This study utilized three independent transcriptomic datasets, including GSE140146 (sustained hypoxia exposure in right ventricle), GSE129848 (hypoxia exposure during a critical prenatal window, encompassing E21 fetal rat hearts and hearts from adult offspring), and GSE133402 (subacute hypoxia exposure in adult left/right ventricle), to systematically compare molecular response signatures under various exposure paradigms (sustained hypoxia, prenatal programming effects, and subacute adaptation) through differential expression analysis of hypoxia-responsive genes. Specifically, this research integrated core circadian gene sets for co-expression pattern analysis, focusing on dissecting the specific remodeling patterns within the circadian rhythm network under sustained hypoxia and its interactions with metabolic-immune pathways. To mitigate potential confounding influences arising from unknown sampling times, robust validation of differential expression and correlation results was performed using random permutation tests. Subsequently, a multi-tiered enrichment analysis framework (including overall transcriptome GSEA pathway analyses, pathway enrichment specific to circadian DEGs, disease association enrichment, and cardiovascular drug target enrichment) was employed. This approach systematically revealed the molecular mechanisms underlying sustained hypoxic adaptation and to evaluate, across the dimensions of pathways, diseases, and drug targets, whether the metabolic-immune adaptation system and the circadian regulatory system exhibit distinct or convergent response patterns under sustained hypoxia. The findings are anticipated to provide novel theoretical foundations for the prevention and treatment of hypoxia-associated heart disease in populations like high-altitude residents and suggest potential targets for therapies targeting metabolic pathways.

## 2. Materials and methods

### 2.1 Data preparation and dataset group definition

RNA-Seq raw data pertaining to cardiac hypoxia studies were retrieved from the NCBI GEO SRA database, encompassing the following datasets: (1) **GSE140146** (sustained hypoxia-exposed group, samples derived from the right ventricle of adult rats continuously maintained in a hypobaric hypoxia chamber (simulating ~2743 m (9000 ft) altitude) from fertilization to 6 weeks of age [n = 14 hypoxia-exposed; n = 11 normoxic controls]), (2) **GSE129848** (Critical prenatal window hypoxia-exposed group, comprising two developmental timepoints: (i) whole heart samples from E21 fetuses immediately after maternal hypoxia exposure (10% O_2_) from E15 to E21 [n = 3 hypoxia-exposed fetuses; n = 3 normoxic control fetuses], (ii) whole heart samples from adult offspring maintained under normoxic conditions until 5 months postnatal age following identical in utero hypoxia exposure [n = 10 hypoxia-exposed; n = 10 normoxic controls]), and (3) **GSE133402** (Adult subacute hypoxia-exposed group, samples obtained from adult rats after 2-week exposure to normobaric hypoxia (10% O_2_). Differential gene expression analysis was performed separately for left ventricle (LV) and right ventricle (RV) tissues [LV hypoxia: n = 3, LV control: n = 3; RV hypoxia: n = 3, RV control: n = 3]).

Raw sequence reads underwent quality assessment and preprocessing. Subsequently, alignment to the *Rattus_norvegicus* reference genome GRCr8.114 (annotation file: `Rattus_norvegicus.GRCr8.114.gtf`) was performed using featureCounts (part of the Subread package) to generate raw counts for each gene [14]. Count matrices from the individual datasets were filtered to retain genes exhibiting raw counts ≥10 in at least 3 samples. Gene annotation was then applied utilizing the “rnorvegicus_gene_ensembl” dataset via the biomaRt package within the R statistical environment [15,16].

These 3 datasets were organized into 5 independent transcriptomic gene count matrices: Sustained Hypoxia RV (GSE140146, n = 25), Prenatal Hypoxia Fetal Hearts (GSE129848 fetuses, n = 6), Prenatal Hypoxia Adult Programmed Hearts (GSE129848 adults, n = 20), and two matrices for Acute Subacute Hypoxia Ventricles (GSE133402 RV [n = 6] and LV [n = 6]).

### 2.2 Transcriptome differential expression analysis

To investigate hypoxia-induced cardiac adaptive changes in developmental and adult models, the 5 transcriptomic count matrices underwent uniform pre-processing. This included removal of duplicate genes (retaining the isoform with the highest expression), conversion to integer values, and quality control filtering (retaining genes with ≥10 counts in ≥3 samples). Subsequently, differential expression analysis was performed separately on each matrix using DESeq2 [17]. Condition-responsive genes were identified based on a negative binomial model (Wald test with FDR correction). Full results were archived for downstream analyses.

### 2.3 Analysis of stage-specific hypoxic effects on the cardiac transcriptome

Full DESeq2 results from the following five groups were filtered based on |log_2_Fold Change (FC)| > 1 and False Discovery Rate (FDR) < 0.05 to identify hypoxia-responsive differentially expressed genes (DEGs) for each group: Sustained Hypoxia RV, Prenatal Hypoxia Fetal Hearts, Prenatal Hypoxia Adult Programmed Hearts, Subacute Hypoxia RV, and Subacute Hypoxia LV. For the subacute hypoxia group (GSE133402): the common RV/LV hypoxia-response DEGs were obtained by intersecting the RV-DEGs and LV-DEGs. The final DEG set for this group was defined as the union of RV-DEGs and LV-DEGs. These DEG sets were used for systematic cross-comparison across the three distinct hypoxia exposure paradigms: lifelong adaptation (sustained hypoxia cohort), developmental critical window (prenatal hypoxia-exposed E21 fetuses), and acute adult adaptation (adult subacute hypoxia exposure). This aimed to identify core conserved hypoxia-responsive genes involved in cardiac adaptation.

### 2.4 Analysis of hypoxic effects on circadian gene expression and function

Reference circadian rhythm genes were first obtained from the Rat Genome Database (RGD) [18,19]. Subsequently, clusterProfiler was employed to comprehensively retrieve all genes associated with the Gene Ontology term GO:0007623 (“circadian rhythm”) and its descendant terms for Rattus norvegicus. Integration and cross-validation of RGD and GO annotations yielded a Core Circadian Gene Set and an Expanded Circadian Gene Set. The high-confidence Core Set comprised genes present in both the RGD collection and the GO pathway annotations. The Expanded Set included genes annotated only by the GO pathway or exclusively recorded in the RGD circadian rhythm collection. These integrated circadian gene sets were then intersected with the DEGs identified in Section 2.3 under the three distinct hypoxia conditions, yielding the hypoxia-responsive circadian DEGs (circadian DEGs).

Pearson correlation analysis was performed to examine the interrelationships among circadian DEGs within the Sustained Hypoxia group. These circadian DEGs were then correlated against non-circadian DEGs to identify circadian-associated interaction partners based on strong co-expression (Pearson |r| > 0.8, FDR < 0.05). To analyze the stability of interactions involving circadian genes (designated as core genes), we identified circadian-associated partners under both normoxia and hypoxia conditions. The overall interaction strength within each condition was quantified by calculating the mean absolute correlation coefficient (mean |r|) among the circadian-associated interaction partners. To assess the robustness of Pearson correlations, Spearman rank correlation was also calculated for all gene pairs, and 95% confidence intervals were computed using Fisher's z-transformation. Leave-one-out sensitivity analysis was performed by iteratively removing each sample and recalculating correlations to identify gene pairs potentially driven by individual observations.

### 2.5 Robustness validation of differential expression and correlations

Given the unknown exact sampling times during the light:dark cycle, potential circadian fluctuations could theoretically confound the differential expression and correlation analyses. To assess whether the observed changes could be attributed to random sampling variation rather than genuine hypoxia effects, a permutation-based validation approach was implemented [20]. We note that this approach evaluates statistical robustness against random label assignment but cannot substitute for recorded sampling time information, a limitation shared by all circadian analyses using publicly available transcriptomic data.

For differential expression analysis, a permutation test was performed on the 8 core circadian genes (*Ass1*, *Bmal1*, *Drd4*, *Npas2*, *Per3*, *Adora2a*, *Id1*, and *Per2*). Sample group labels (hypoxia *vs.* control) were randomly shuffled 10,000 times, and a t-test was recalculated for each permutation. An empirical p-value was computed as the proportion of permutations producing a p-value less than or equal to the observed p-value. A low empirical p-value would indicate that the differential expression cannot be explained by random sampling variation.

For correlation analysis, two complementary permutation approaches were employed. First, within-group correlation robustness was assessed by randomly permuting sample labels within each experimental group (*e.g*., hypoxia group only) while preserving the expression matrix, repeating this 10,000 times to estimate the probability of observing the actual correlation coefficient by chance. Second, between-group correlation difference was evaluated by randomly permuting group assignments across all samples to construct a null distribution of correlation differences (Δr = r_hypoxia_ – r_control_). The empirical p-value was calculated as the proportion of permutations yielding a |Δr| greater than or equal to the observed value. Gene pairs with empirical p-values < 0.05 were considered robust and unlikely to arise from circadian rhythm-related sampling artifacts. The analysis focused on two categories of gene pairs: (1) core circadian gene pairs showing significant correlation changes between groups, and (2) interaction pairs between core circadian genes and differentially expressed genes that exhibited statistically significant correlation shifts (Fisher's z test p < 0.05).

### 2.6 Elucidation of Cross-species Molecular Mechanisms Underlying Sustained Hypoxic Adaptation

To explore differences in hypoxia-response patterns, significantly enriched pathways among the Sustained Hypoxia RV, Subacute Hypoxia LV/RV, and Prenatal Hypoxia Adult Programmed Hearts groups were compared. To systematically uncover molecular adaptation mechanisms in the RV under sustained hypoxia, a multi-tiered enrichment analysis framework was applied to the full DESeq2 differential expression results (ranked gene list based on log_2_ fold change (log_2_FC)) for the Sustained Hypoxia RV dataset. The initial step involved mapping the 18,452 rat genes to their human orthologs using the RGD ortholog mapping file “RGD_ORTHOLOGS.txt” (Version 2.1.6, released 2020/01/10) [18,19] to enable cross-species pathway analysis.

In the first tier, Gene Set Enrichment Analysis (GSEA) employing gseKEGG was applied to scan the KEGG pathway database comprehensively, identifying pathway modules exhibiting coordinated changes at a significance threshold of FDR < 0.05. The second tier focused specifically on the hypoxia-responsive circadian DEGs identified in the Sustained Hypoxia group; KEGG pathway enrichment analysis, performed using enrichR against the KEGG_2021_Human database, revealed significantly enriched pathways (FDR < 0.05) associated with these rhythm-implicated genes. Finally, the enriched pathways identified in the whole transcriptome GSEA (Tier 1) and the circadian DEG-specific enrichment (Tier 2) were systematically compared to identify overlapping pathway sets. This comparative integration evaluated the potential association between circadian rhythm regulation and broader transcriptomic changes under sustained hypoxia.

### 2.7 Disease enrichment analysis

Core pathway genes identified by gseKEGG analysis (Section 2.6) and hypoxia-responsive circadian DEGs (Section 2.4) underwent disease enrichment analysis using the DOSE package, based on mapped human orthologs. To prioritize cardiovascular-relevant findings, a multi-step filtering strategy was applied: 1) Keyword screening against a list of 26 cardiac diseases (e.g., “heart failure,” “hypertension,” “cardiomyopathy”); 2) Exclusion of terms related to neoplasms and other non-relevant diseases. A composite score, integrating enrichment significance strength and clinical relevance, was calculated to quantify pathway-disease associations.

### 2.8 Cardiovascular disease-related drug target analysis

Based on the cardiac disease-associated pathways and their mapped genes identified in Section 2.7, drug target enrichment analysis was performed separately for both analytical tiers using enrichR. For Tier 1 (core pathway genes from gseKEGG), genes mapped to cardiac disease-related pathways as identified in Section 2.7 were extracted for drug target analysis. For Tier 2 (hypoxia-responsive circadian DEGs), the entire DEG list was analyzed for drug target enrichment, with results then filtered for cardiovascular-relevant drugs. Four specialized drug-gene target interaction databases (DGIdb Drug Targets 2024, IDG Drug Targets 2022, DrugBank, and DSigDB) were queried. Significance was set at FDR < 0.05. To focus on cardiovascular therapeutic drugs, hits were further screened via keyword matching using relevant terms: “beta blocker,” “calcium channel blocker,” “alpha blocker,” “diuretic,” “vasodilator,” “anti-arrhythmic” (e.g., “amiodarone,” “adenosine”), “inotropic agent” (e.g., “digoxin,” “dopamine”), “anti-anginal” (e.g., “ranolazine,” “ivabradine,” “trimetazidine”), “antiplatelet”/“anticoagulant”(e.g., “aspirin,” “warfarin”), “lipid-lowering” (e.g., “statin”) and “metabolic modulator” (“etomoxir,” “perhexiline,” “propionyl-L-carnitine”).

### 2.9 Statistical analysis

For all analyses described in this study, unless explicitly stated otherwise in the preceding methodological sections, standard statistical parameters were applied. Significance thresholds were universally set at a false discovery rate (FDR) < 0.05. Differentially expressed genes (DEGs) were identified using a filtering criterion of absolute log_2_ fold change (|log_2_FC|) > 1. Co-expression relationships revealed by Pearson correlation analysis were considered biologically significant if the absolute correlation coefficient (|r|) exceeded 0.8. Robustness assessments via permutation testing employed 10,000 random iterations to generate empirical p-values. Functional and disease enrichment analyses were conducted exclusively using genes mapped to their human orthologs from the rat transcriptome data. All statistical computations and data manipulations were executed within the R statistical computing environment, version 4.3.1. Core analyses utilized the following R/Bioconductor packages: differential expression analysis with DESeq2; functional enrichment analyses using clusterProfiler and enrichR; disease enrichment analysis with DOSE; data processing and visualization via tidyverse; and gene annotation facilitated by org.Rn.e.g.,db (rat) and org.Hs.e.g.,db (human) annotation databases. For correlation analyses, p-values were adjusted using the Benjamini-Hochberg false discovery rate (FDR) correction. Given the exploratory nature of the co-expression network analysis and the relatively small sample size, we report both FDR-corrected and nominal p-values where appropriate, and acknowledge that Bonferroni correction would be overly conservative for the number of gene pairs tested.

## 3. Results

### 3.1 Establishment of sustained hypoxia as the core focus

**Fig 1:**
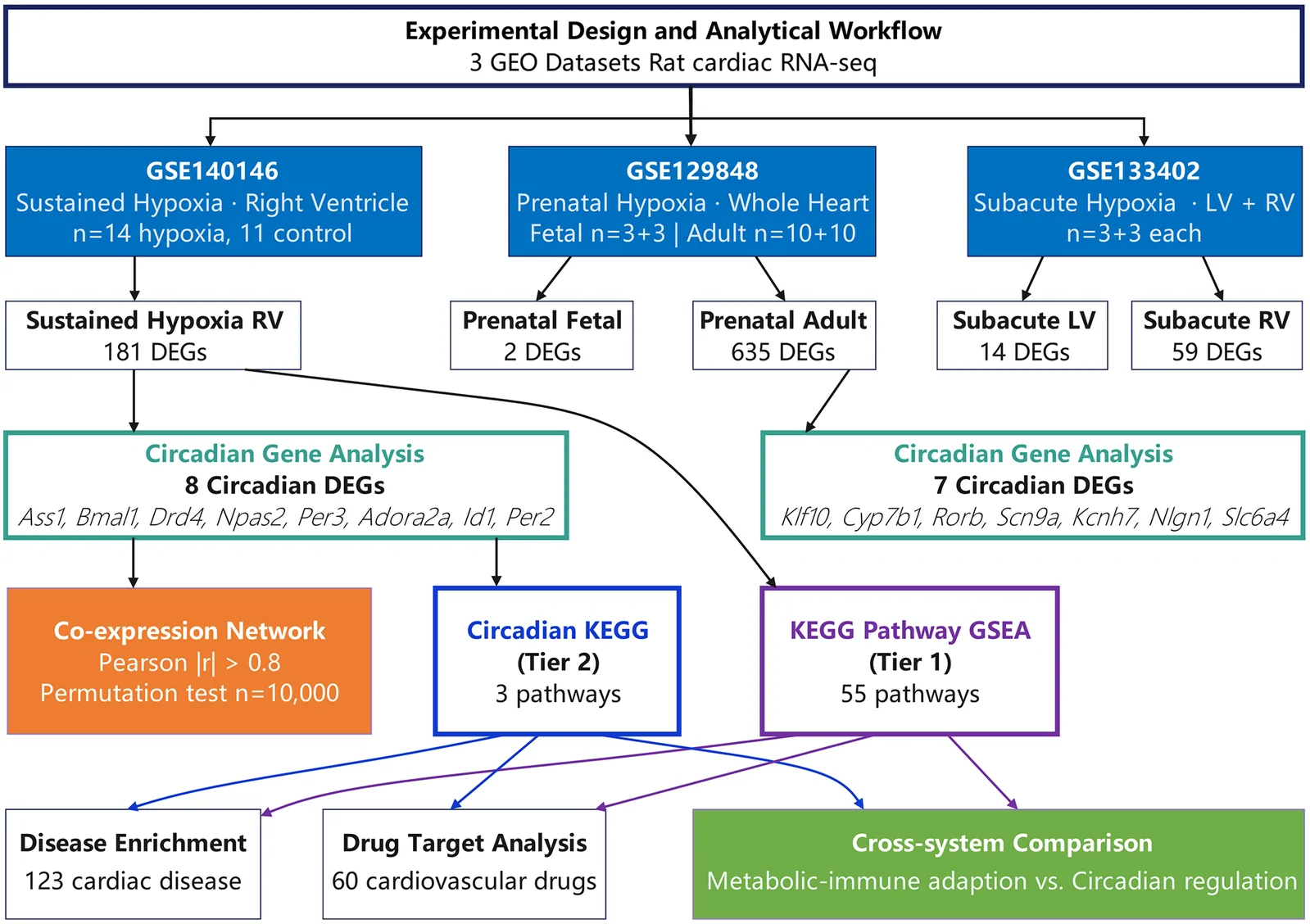
Experimental design and analytical workflow. Three GEO datasets (GSE140146, sustained hypoxia; GSE129848, prenatal hypoxia; GSE133402, subacute hypoxia) were organized into five transcriptomic count matrices. Differential expression analysis (DESeq2, |log_2_FC| > 1, FDR < 0.05) identified DEGs across all five groups. Circadian gene sets from RGD and GO databases were intersected with DEGs to identify hypoxia-responsive circadian DEGs (8 in sustained hypoxia, 7 in prenatal adult). Co-expression network analysis (Pearson |r| > 0.8, permutation test n = 10,000) was performed on the sustained hypoxia group. A multi-tiered enrichment framework including KEGG pathway GSEA (Tier 1), circadian gene KEGG enrichment (Tier 2), disease enrichment, and drug target analysis was applied, culminating in cross-system comparison of metabolic-immune adaptation versus circadian regulation.

The overall experimental design and analytical workflow are illustrated in Fig 1. Differential expression analysis using DESeq2 revealed 181 differentially expressed genes (DEGs) in the Sustained Hypoxia group (sustained-DEGs), indicating substantial transcriptomic alterations induced by chronic hypoxia. In contrast, E21 fetal hearts exposed to hypoxia during the critical prenatal window (E15-E21) exhibited only 2 DEGs (prenatal fetal-DEGs), though the limited sample size (n = 3 per group) constrains the statistical power to detect smaller transcriptional changes. In the Adult Subacute Hypoxia group, ventricular compartment-specific analysis identified 59 DEGs in the right ventricle (RV-DEGs) and 14 DEGs in the left ventricle (LV-DEGs), with their union constituting 67 unique Adult Subacute-DEGs. Notably, six overlapping genes (*Ccdc71*, *Ppfia4*, *Tceal7*, *E2f7*, *Cdc42bpa*, *Gsta4*) identified from the RV/LV intersection represent common hypoxia-responsive elements across both ventricles. Crucially, cross-comparison among all four DEG sets revealed no shared transcript between the sustained-DEGs, prenatal fetal-DEGs, and Adult Subacute-DEGs. In the Prenatal Adult Programmed Heart group (GSE129848 adult offspring exposed to prenatal hypoxia E15–E21 and subsequently raised under normoxia to 5 months of age), 635 DEGs were identified (|log_2_FC| > 1, FDR < 0.05; Supplementary S2 Table). Only 13 DEGs (1.6%) overlapped with the sustained hypoxia group, and a single gene (Acta1) was shared with the adult subacute group. No DEGs were shared with the prenatal fetal group. This limited overlap among the four DEG sets likely reflects a combination of genuine biological divergence and experimental design heterogeneity (Supplementary S2 Table). Given the distinct transcriptional signature and the unique lifelong exposure paradigm, as opposed to the prenatal programming model of the adult offspring group, we designated the sustained hypoxia group as the core focus for subsequent in-depth analyses.

### 3.2 Specific remodeling of the circadian interactome by sustained hypoxia

**Fig 2:**
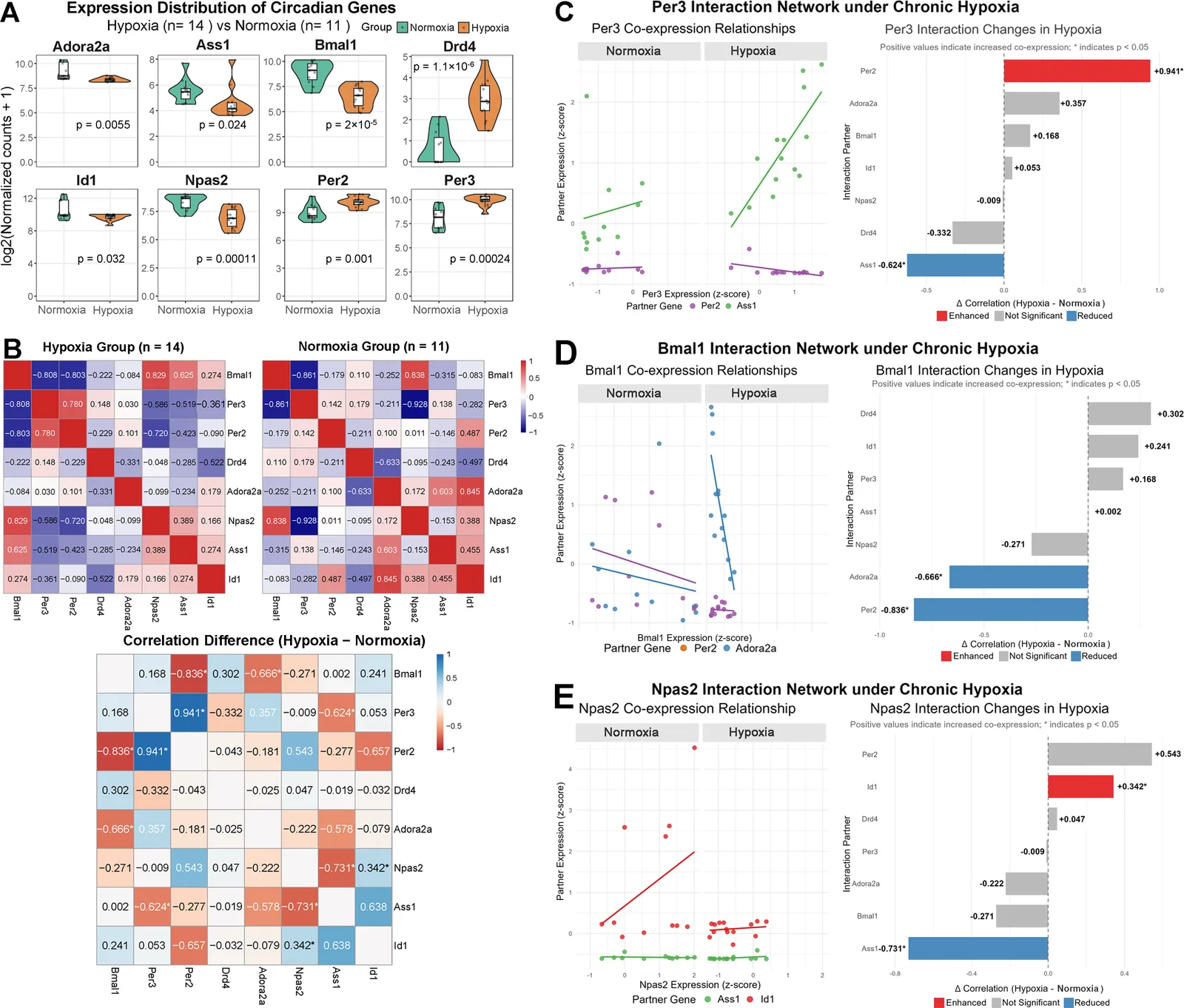
Sustained Hypoxia Induces Hub Gene Transition and Evolution of Interaction Strength in the Core Circadian Network. (A) Violin plots illustrating the expression levels of eight clock differentially expressed genes (Ass1, Bmal1, Drd4, Npas2, Per3, Adora2a, Id1, and Per2) in the control (normoxia) group and the sustained hypoxia group. The dashed line indicates the median expression level in the control group, demonstrating the expression changes of these clock differentially expressed genes under sustained hypoxia. (B) Comparative analysis of co-expression patterns among the eight clock differentially expressed genes. The left panel displays a pairwise Pearson correlation heatmap matrix for the eight circadian rhythm-related genes in the hypoxia group (n = 14), while the right panel shows the corresponding correlation heatmap for the normoxic control group (n = 11). The lower panel presents the differential correlation (r_hypoxia − r_normoxia) heatmap. Red indicates positive correlations, and blue indicates negative correlations; asterisks denote statistically significant differences (p < 0.05). (C–E) Dynamic changes in interaction networks for *Per3*, *Bmal1*, and *Npas2* genes under hypoxic stress, respectively. The left panels show standardized expression scatter plots (z-score), with both axes representing z-score-normalized expression values. The right panels display interaction change bar charts (sorted by Δr), where positive bars indicate enhanced synergy under hypoxia and negative bars indicate weakened synergy. Asterisks mark statistically significant changes.

Based on this foundation, we next examined the impact of sustained hypoxia on the circadian rhythm system. Integration and cross-validation of the Rat Genome Database (RGD) and Gene Ontology (GO) annotations yielded 148 core circadian genes and 133 expanded circadian genes. Differential expression analysis (Fig 2A) revealed 8 hypoxia-responsive circadian DEGs in the Sustained Hypoxia group: 5 core genes (*Ass1*, *Bmal1*, *Drd4*, *Npas2*, *Per3*) and 3 expanded genes (*Adora2a*, *Id1*, *Per2*). No significant circadian DEGs were detected in the Prenatal Hypoxia fetal group or the Adult Subacute Hypoxia group (including both RV and LV subgroups). In the Prenatal Adult Programmed Heart group, 7 circadian DEGs were identified (*Klf10*, *Cyp7b1*, *Rorb*, *Scn9a*, *Kcnh7*, *Nlgn1*, *Slc6a4*), none of which overlapped with the 8 circadian DEGs from the sustained hypoxia group, suggesting that the developmental timing and duration of hypoxia exposure may influence which circadian genes are affected, though differences in tissue type and age at sampling could also contribute.

We subsequently employed Pearson correlation analysis to systematically profile the remodeling of the circadian interactome under sustained hypoxia conditions. Our analysis revealed a striking finding: the total number of significant rhythm gene-pairs (Pearson |r| > 0.8, FDR < 0.05) plummeted by 67.5%, decreasing from 160 pairs in normoxia to merely 52 pairs under hypoxia. More critically, hypoxia triggered a systematic rewiring of the core clock subnetwork (Fig 2B), which manifested in three distinct adaptive interaction patterns among the clock differentially expressed genes.

The first pattern, termed enhanced synchronicity, was characterized by the strengthening of existing positive or negative correlations between specific clock elements (Fig 2C). The positive correlation between *Per2* and *Per3* was significantly strengthened under hypoxia conditions (r = 0.780 *vs.* 0.142, p = 0.022). Simultaneously, the inhibitory interaction between *Bmal1* and *Per2* strengthened nearly four-fold, shifting from r = −0.179 in normoxia to r = −0.803 under hypoxia (p = 0.044). The negative correlation between *Bmal1* and *Adora2a* also changed significantly (p = 0.023), though the interaction remained weak. The second pattern involved complete reversals in the direction of gene-gene correlations (Fig 2D). The correlation between *Per3* and *Ass1* inverted from a weak positive association (r = 0.138) in normoxia to a strongly negative correlation (r = −0.519) in hypoxia (p = 0.046). Similarly, the correlation between *Npas2* and *Ass1* reversed from a negative relationship in normoxia (r = −0.153) to a positive correlation under hypoxia (r = 0.389; p = 0.048). The third pattern, described as weakened synchronicity, encompassed significant reductions in correlation strength between interacting partners (Fig 2E). The positive correlation between *Npas2* and *Id1* weakened by approximately 57%, shifting from r = 0.388 in normoxia to r = 0.166 under hypoxia (p = 0.036). The strong positive correlation between *Adora2a* and *Id1* observed in normoxia (r = 0.845) was drastically weakened to near negligible levels under hypoxia (r = 0.179), though this particular change did not achieve statistical significance (p = 0.849). Notably, despite these substantial network-level alterations, the core oscillator interaction between *Bmal1* and *Npas2* remained remarkably stable, with r-values of 0.838 in normoxia and 0.829 under hypoxia (Δr = −0.009), indicating that the fundamental core clock machinery maintains a degree of resilience against hypoxic stress despite the overall remodeling of the network.

**Fig 3:**
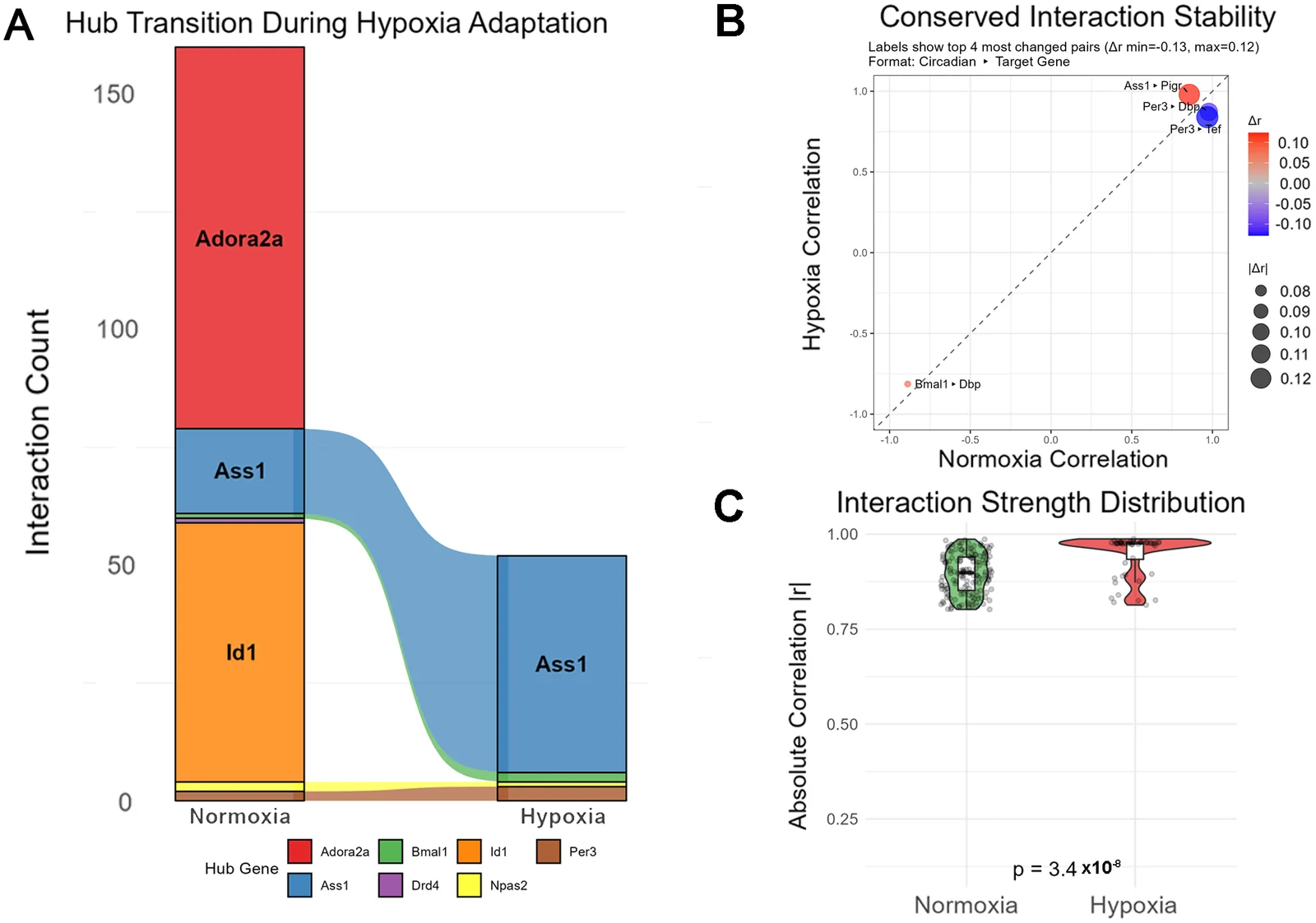
Hub Gene Transition and Rewiring of Interaction Intensity in the Core Circadian Network under Sustained Hypoxia. (A) A Sankey diagram intuitively illustrates the transition of hub genes among core circadian genes in response to hypoxic challenge. The left panel represents interaction hub genes under normoxic conditions, while the right panel depicts hub genes in the hypoxic environment. Each flowing ribbon tracks the interaction count of an individual gene (indicated by the same color) during the normoxia-to-hypoxia transition. (B) A correlation plot displaying rhythm-related gene interactions that are significantly present in both normoxic and hypoxic environments (n = 4 pairs). Each point represents a gene interaction pair, with point size corresponding to |Δr| (|r_hypoxia_ − r_normoxia_|), where larger values indicate greater cross-environment changes in interaction strength. Point color indicates the direction of Δr change (Δr > 0 indicates enhanced interaction under hypoxia, shown in red; Δr < 0 indicates weakened interaction, shown in blue; gray indicates no change). (C) Violin plots illustrating the distribution characteristics of gene interaction intensity under both oxygen conditions. The expanded regions of the violin contours (green for normoxia, red for hypoxia) reflect data distribution density, with contour width indicating the density of the distribution. The Y-axis represents absolute correlation values |r| (ranging from 0 to 1), where higher values indicate stronger correlations.

The hypoxic remodeling of the circadian interaction network centered around DEGs was also evident in hub gene reorganization (Fig 3A). Under normoxia, a dual-hub network architecture predominated, with *Id1* (55 targets) and *Adora2a* (81 targets) emerging as the two most prominent hub genes. The metabolism-linked *Ass1* ranked third with 18 targets (11.25% of 160 significant interaction pairs), while the remaining four circadian genes (*Per3*, *Npas2*, *Bmal1*, and *Drd4*) had substantially fewer interaction targets, appearing as peripheral connections in the network visualization. Hypoxia, however, eradicated all significant interactions mediated by *Id1* and *Adora2a*, while dramatically elevating *Ass1*’s dominance to 88.5% (46/52 targets) of all significant pairs. As shown in Fig 3B, across both oxygen conditions, only four gene pairs exhibited significant interactions in common. Specifically, the *Ass1*-*Pigr* co-expression strength significantly increased under hypoxia (Δr = +0.123), whereas the associations of *Per3* with *Tef* and *Dbp* notably weakened in the hypoxic state (Δr = −0.128 and Δr = −0.105, respectively). The *Bmal1*-*Dbp* interaction showed only a modest enhancement (Δr = +0.074).

Furthermore, systematic restructuring of gene interaction strength was observed under hypoxic stress (Fig 3C). Comparative analysis of the absolute correlation coefficient (|r|) distributions for significantly correlated gene pairs (p < 0.05) revealed a striking shift: the hypoxia group exhibited a median |r| of 0.975 (IQR: 0.976–0.980), representing a 5.2% increase compared to the normoxia group (median = 0.927, IQR: 0.899–0.954; independent samples t-test: t = −10.32, p < 0.001). The hypoxic distribution assumed a “highly peaked” morphology, with 88.5% of interaction pairs concentrated in the |r| > 0.97 interval, completely eliminating weak associations below |r| = 0.8. In stark contrast, the normoxic group retained a broader tail of weaker correlations, with 95% of significant pairs still exceeding |r| = 0.8. Representative exemplary cases include the *Ass1*-*Pigr* interaction, where |r| increased from 0.856 in normoxia to 0.980 in hypoxia (a 14.5% increase), and the *Per3*-*Dbp* interaction, where |r| shifted from 0.977 in normoxia to 0.872 in hypoxia. To assess the robustness of these Pearson correlations, Spearman rank correlation was also calculated. While the core oscillator pair Arntl-Npas2 remained consistent across both methods (Spearman ρ = 0.879 in hypoxia, 0.918 in normoxia), the overall Spearman coefficients were substantially lower than Pearson coefficients (median |ρ| = 0.357 vs. median |r| = 0.976 in hypoxia; Supplementary S3 Table), suggesting that the high Pearson correlations may be partly influenced by distributional properties or influential observations. Leave-one-out sensitivity analysis further confirmed that several Ass1-centric gene pairs were sensitive to single-sample removal (Supplementary S3 Table).

### 3.3 Robustness validation of circadian gene expression changes under sustained hypoxia

**Fig 4:**
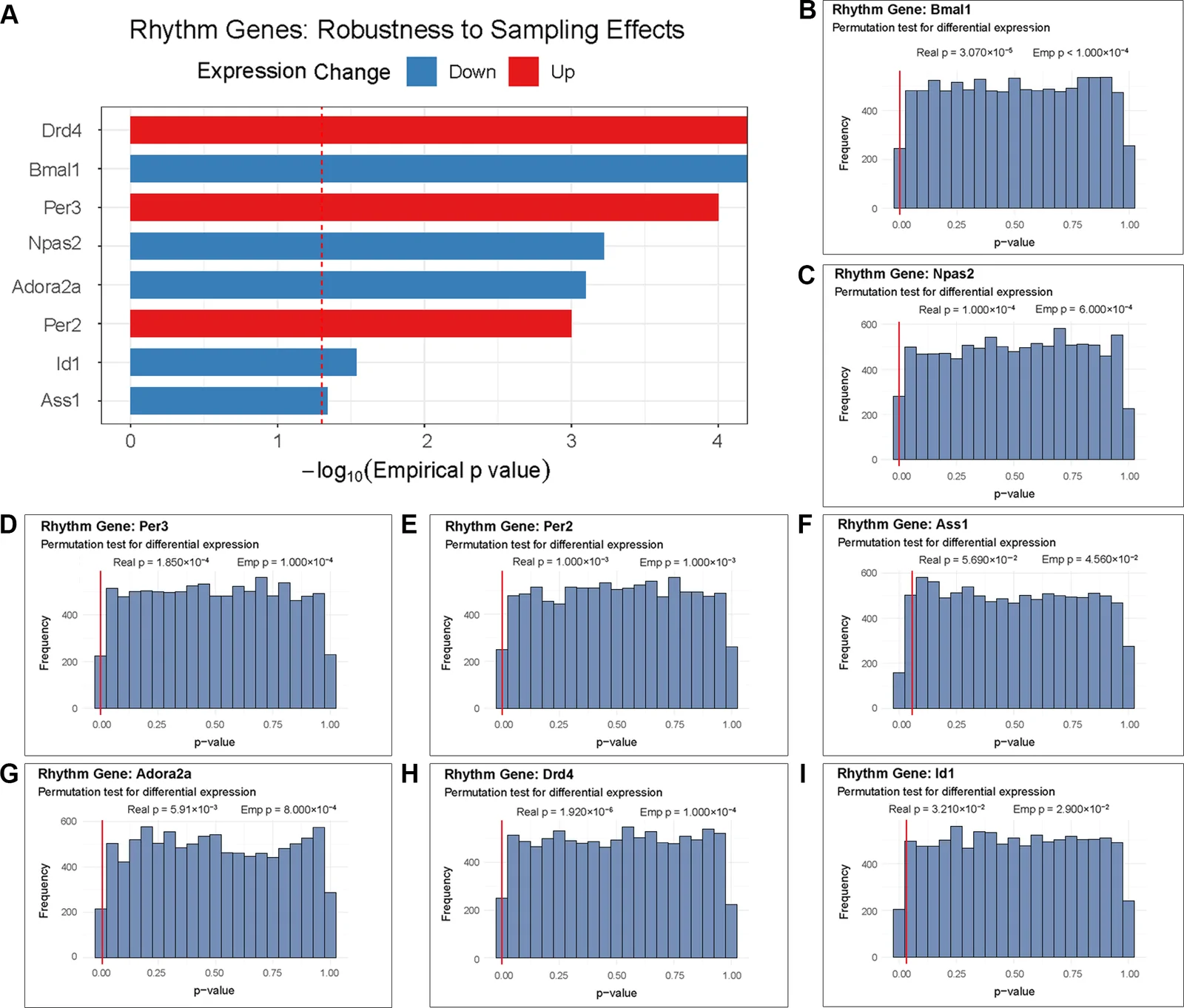
Robustness Validation of Rhythm Gene Hypoxia-Responsive Differential Expression against Sampling Time Variation. (A) A histogram presenting a summary of permutation test results for rhythm-related differentially expressed genes. Genes are sorted by empirical p-value (from most significant to least significant), and the red dashed line indicates the empirical p = 0.05 significance threshold. Blue bars represent downregulated genes, and red bars represent upregulated genes. All eight core circadian genes passed permutation testing validation (empirical p < 0.05), demonstrating their resistance to sampling time variation. (B–I) Histograms showing permutation test distribution for each rhythm-related differentially expressed gene. Each subpanel illustrates the characteristics of the permutation test results for an individual gene. Blue histogram bars represent the frequency density of p-value distributions, where bar height indicates the proportion of 10,000 permutation results falling within each p-value interval. The red vertical line marks the position of the true between-group difference p-value for that gene.

To evaluate the robustness of circadian gene expression changes against random sampling variation, we performed permutation testing to validate the transcriptional signatures of circadian DEGs in the sustained hypoxia RV (Fig 4A). The core regulator *Bmal1* (significantly downregulated, nominal p = 3.067 × 10^−5^) completely resisted random sampling effects, its significance was unmatched in 10,000 permutations (empirical p < 1.000 × 10^−4^). This robustness extended to all other core clock elements exhibiting sustained hypoxia specific dysregulation (Fig 4B –4I), including *Ass1* (downregulated, nominal p = 5.691 × 10^−2^, empirical p = 4.560 × 10^−2^), *Adora2a* (downregulated, nominal p = 5.907 × 10^−3^, empirical p = 8.000 × 10^−4^), *Drd4* (upregulated, nominal p = 1.920 × 10^−6^, empirical p < 1.000 × 10^−4^), *Id1* (downregulated, nominal p = 3.205 × 10^−2^, empirical p = 2.900 × 10^−2^), *Npas2* (downregulated, nominal p = 1.005 × 10^−4^, empirical p = 6.000 × 10^−4^), *Per2* (upregulated, nominal p = 1.004 × 10^−3^, empirical p = 1.000 × 10^−3^), and *Per3* (upregulated, nominal p = 1.845 × 10^−4^, empirical p = 1.000 × 10^−4^).

At the molecular interaction level, a dual permutation scheme was used to assess the circadian gene co-expression network alterations under hypoxia. For the within-group interaction robustness analysis, we performed 10,000 permutations to assess whether the observed correlations exceeded random expectations. High-fidelity interactions in the sustained hypoxia group included, among others, *Bmal1*-*Tef* (r = −0.884, empirical p < 1.0 × 10^−4^), *Ass1*-*Hpgd* (r = 0.982, empirical p = 4.3 × 10^−3^), *Ass1*-*Pla2g2d* (r = 0.976, empirical p = 2.6 × 10^−3^), and *Per3*-*Per2* (r = 0.780, empirical p = 1.7 × 10^−3^), all showing exceptionally low probability of random occurrence.

**Fig 5:**
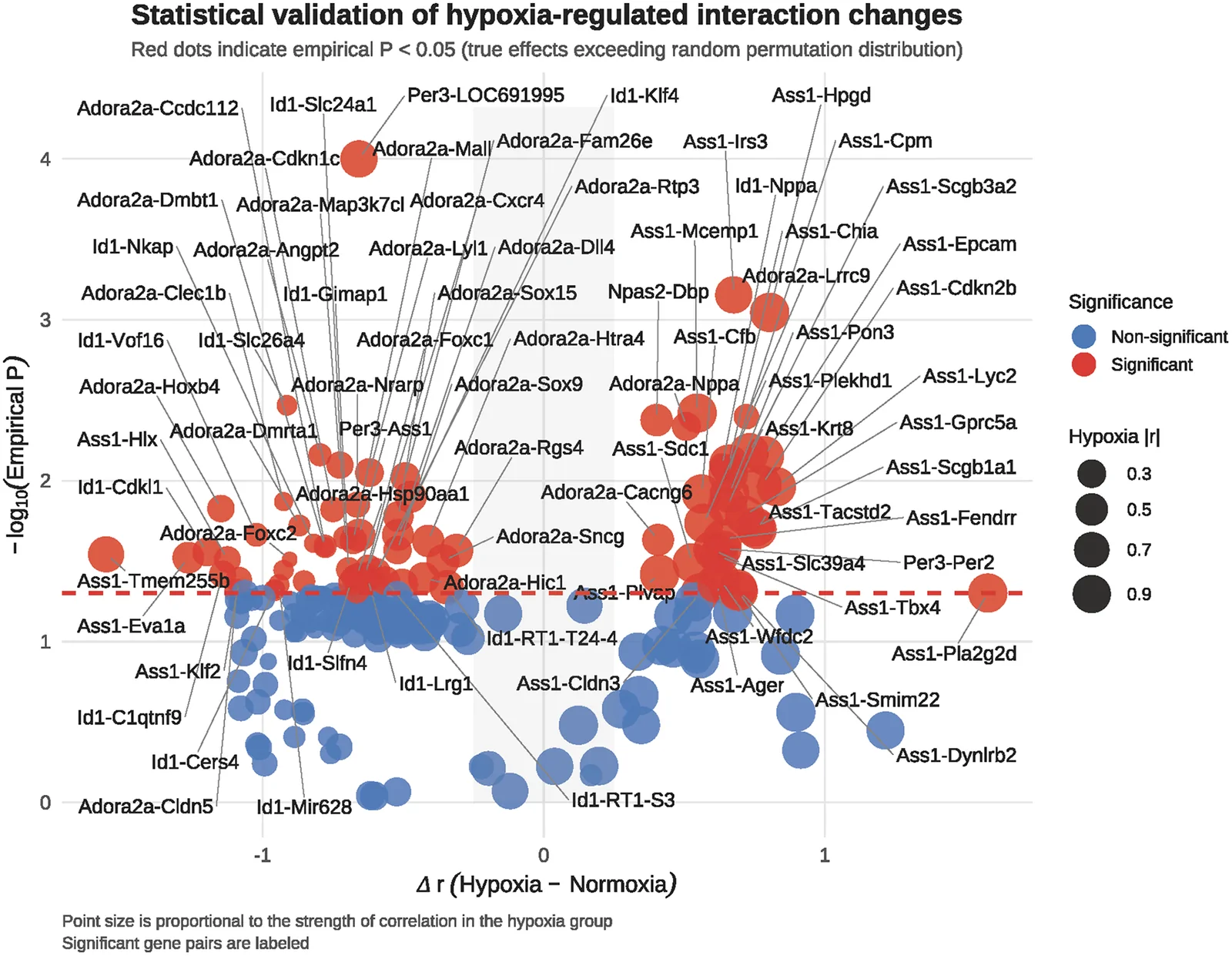
Statistical Validation of Hypoxia-Regulated Molecular Interaction Network Changes. A statistical triangle validation plot illustrates the specific changes in rhythm-related interaction genes under chronic hypoxic conditions. Each point represents a gene pair. The horizontal axis (Δr) represents the difference between the correlation coefficient in the hypoxia group (r) and that in the normoxia group (r), with positive values indicating enhanced interaction under hypoxia. The vertical axis represents the negative logarithm of the empirical p-value (−log_10_), with the dashed line indicating the statistical significance threshold (p = 0.05). Point size corresponds to the absolute value of the correlation coefficient in the hypoxia group (|r|), where larger points indicate stronger interactions in the hypoxia group. Points colored in red indicate empirical p < 0.05 (significant hypoxia-specific changes), while blue points indicate non-significant changes. The gray shaded region (|Δr| < 0.25) represents biologically insignificant changes that may be influenced by sampling variability.

Between-group interaction difference analysis identified 85 significantly altered gene pairs (empirical p < 0.05), representing 40.3% of the 211 tested pairs. These changes were predominantly mediated by three core circadian genes: *Ass1* (31 significant pairs), *Adora2a* (31 significant pairs), and *Id1* (18 significant pairs), collectively accounting for 94% of all significant network rewiring. The magnitude of correlation changes was substantial, with a mean |Δr| of 0.72 (median = 0.67, range = 0.31–1.58). Notably, 52 pairs showed weakened correlations (Δr < 0) while 33 pairs showed strengthened correlations (Δr > 0) in hypoxia, suggesting that chronic hypoxia preferentially disrupts existing co-expression relationships over establishing new ones (Fig 5). Representative examples of hypoxia-specific rewiring included: *Ass1*-*Mcemp1* (r: 0.438 to 0.983, Δr=+0.546, empirical p = 3.8 × 10^−3^), *Id1*-*Krt8* (r: 0.984 to 0.345, Δr = −0.639, empirical p = 2.2 × 10^−2^), and *Adora2a*-*Map3k7cl* (r: 0.964 to 0.239, Δr = −0.725, empirical p = 8.0 × 10^−3^). In contrast, the hub interaction between *Ass1* and *Pigr* remained highly stable across groups (r = 0.980 in hypoxia vs. 0.856 in normoxia, Δr = 0.12, empirical p = 0.33), confirming the presence of core hypoxia-resistant circadian modules. These results indicate that the observed circadian gene expression changes and networks alterations are unlikely to be explained by random sampling variation alone. However, because actual sampling times were not recorded in the original studies, we cannot exclude the possibility that systematic differences in time of tissue collection between hypoxia and control groups contributed to the observed circadian gene differences.

### 3.4 Divergent pathway enrichment profiles of metabolic-immune and circadian system under sustained hypoxia

We further employed a two-tiered KEGG pathway enrichment strategy to dissect the cross-species molecular response landscape of the heart under sustained hypoxic exposure.

In the first tier, Gene Set Enrichment Analysis was performed on the whole transcriptome of the sustained hypoxic right ventricle. This analysis identified 55 significantly enriched KEGG pathways at an FDR threshold of 0.05 (Supplementary S1 Table). These pathways exhibited a distinct bidirectional regulation pattern. At the metabolic level, energy-related pathways, including oxidative phosphorylation (NES = 2.45, FDR = 3.76 × 10^−7^), carbon metabolism (NES = 2.39, FDR = 6.34 × 10^−7^), and pyruvate metabolism (NES = 2.54, FDR = 8.25 × 10^−6^), were significantly up-regulated. Simultaneously, ancillary metabolic pathways, including citrate cycle (TCA cycle) (NES = 2.53, FDR = 9.16 × 10^−6^), valine, leucine and isoleucine degradation (NES = 2.43, FDR = 9.16 × 10^−6^), propanoate metabolism (NES = 2.40, FDR = 5.74 × 10^−5^), 2-oxocarboxylic acid metabolism (NES = 2.31, FDR = 2.09 × 10^−4^), glyoxylate and dicarboxylate metabolism (NES = 2.29, FDR = 2.23 × 10^−4^), and fatty acid degradation (NES = 2.21, FDR = 5.53 × 10^−4^), were uniformly up-regulated. These observations collectively suggest that cardiomyocytes optimize energy supply through multi-pronged substrate utilization under sustained hypoxia. Pathways related to myocardial structural and functional capacity, including cardiac muscle contraction (NES = 2.04, FDR = 5.53 × 10^−4^), cytoskeleton in muscle cells (NES = 1.91, FDR = 1.14 × 10^−4^), and thermogenesis (NES = 1.83, FDR = 2.97 × 10^−4^), also demonstrated positive enrichment, implying adaptive enhancement of myocardial contractile function and metabolic regulation. In marked contrast, pathways involved in immune and signaling regulation were predominantly down-regulated. The IL-17 signaling pathway (NES = −1.86, FDR = 2.54 × 10^−3^), cytokine-cytokine receptor interaction (NES = −1.79, FDR = 7.14 × 10^−4^), and NF-κB signaling pathway (NES = −1.68, FDR = 2.35 × 10^−2^) exhibited significant negative enrichment. Similarly, the complement and coagulation cascades (NES = −1.95, FDR = 6.04 × 10^−4^), TNF signaling pathway (NES = −1.64, FDR = 2.88 × 10^−2^), and TGF-β signaling pathway (NES = −1.75, FDR = 6.82 × 10^−3^) all displayed negative enrichment. These findings indicate that sustained hypoxia concurrently activates metabolic adaptation mechanisms while inducing an adaptive down-regulation of immune pathways.

The second tier of analysis focused specifically on the eight circadian differentially expressed genes identified in the sustained hypoxia group, namely *Ass1*, *Bmal1*, *Drd4*, *Npas2*, *Per3*, *Adora2a*, *Id1*, and *Per2*. Targeted KEGG pathway enrichment analysis revealed three significantly enriched pathways at FDR < 0.05, which were functionally categorized as circadian rhythm-related modules (hsa04710, hsa04713) and neuroactive ligand-receptor interaction (hsa04728). Cross-comparison between the two enrichment tiers revealed no overlapping pathways between the circadian gene-derived KEGG results and the whole-transcriptome GSEA results. This absence of pathway-level convergence suggests that, under sustained hypoxia, the metabolic-immune adaptation system and the circadian rhythm regulatory system exhibit divergent enrichment profiles at the pathway level, with no overlapping KEGG pathways identified between the two analytical tiers.

### 3.5 Disease enrichment analysis: divergent pathological associations between the two systems

Building upon the pathway enrichment findings, we further conducted disease enrichment analyses to explore the differential pathological associations between the metabolic-immune adaptation system and the circadian regulatory system. Using a stringent significance threshold of FDR < 0.05, the 55 significantly enriched pathways identified through whole-transcriptome GSEA of the sustained hypoxic right ventricle were initially mapped to 6,369 associated diseases. Following keyword-based filtering for cardiac relevance and exclusion of neoplastic and other non-cardiovascular terms, this list was refined to 123 cardiac-related diseases. In parallel, disease association analysis of the KEGG pathways derived from the eight circadian differentially expressed genes initially identified 234 associated diseases; applying the identical filtering criteria reduced this to merely two cardiac-related diseases. Notably, under the strict FDR < 0.05 threshold for both analyses, no cardiac diseases were co-enriched. However, when the second-tier analysis was relaxed to FDR < 0.10, three cardiac diseases became commonly enriched: chronic heart failure, secondary myocardial diseases, and primary cardiomyopathies.

Further detailed scrutiny revealed fundamental differences in the enrichment characteristics of these shared diseases between the two systems. In the GSEA-derived disease enrichment results, chronic heart failure was associated with 13 distinct KEGG pathways, encompassing cardiac-related, and immune/inflammatory pathways (detailed in Table 1). Both secondary myocardial diseases and primary cardiomyopathies were linked to an identical set of 19 KEGG pathways, encompassing cardiomyopathy-related pathways, immune/inflammatory pathways, metabolic pathways, signaling pathways, and neurodegenerative disease pathways (detailed in Table 1), representing a classic multi-pathway, polygenic regulatory network. In stark contrast, in the circadian gene-derived disease enrichment results, chronic heart failure, secondary myocardial diseases, and primary cardiomyopathies all represented single-gene enrichments (specifically ADORA2A), with FDR values exceeding 0.05 and thus failing to meet conventional statistical significance thresholds.

**Table 1:** Shared enriched cardiac diseases between two analytical tiers at FDR < 0.1.

Disease	Analysis Tier	Pathways No.	Pathway Types	Core Genes	FDR
Chronic heart failure	GSEA pathways	13	Cardiac-related, immune/inflammatory (1)	Multiple genes	< 0.05
	Hypoxia-responsive circadian genes	–	–	ADORA2A (single)	0.05 ~ 0.1
Myocardial Diseases, Secondary / Cardiomyopathies, Primary	GSEA pathways	17	Cardiac disease, immune/inflammatory (2), metabolism, signaling	Multiple genes	< 0.05
	Hypoxia-responsive circadian genes	–	–	ADORA2A (single)	0.05 ~ 0.1

**Notes:**

1. Pathway Types Explained:

Cardiac-related: Includes diabetic cardiomyopathy (hsa05415), cardiomyocyte signaling (hsa04261), cardiac muscle contraction (hsa04260) and **atherosclerosis (hsa05418)**;

Cardiac disease: Includes hypertrophic cardiomyopathy (hsa05410), arrhythmogenic right ventricular cardiomyopathy (hsa05412), dilated cardiomyopathy (hsa05414), diabetic cardiomyopathy (hsa05415) and **atherosclerosis (hsa05418)**;

Immune/inflammatory (1): Includes **cytokine interactions** (**hsa04060**, hsa04061), chemokine signaling (hsa04082), **TNF signaling (hsa04668)**, NF-κB signaling (hsa04064), leukocyte migration (hsa04670), **hepatitis C (hsa05160), influenza A (hsa05164) and COVID-19 (hsa05171)**;

Immune/inflammatory (2): Includes **cytokine interaction** (**hsa04060**), IL-17 signaling (hsa04657), **TNF signaling (hsa04668)**, **hepatitis C (hsa05160)**, **influenza A (hsa05164) and COVID-19 (hsa05171)**;

Metabolism: Includes peroxisome (hsa04146), thermogenesis (hsa04714), glucagon signaling (hsa04922) and adipocyte lipolysis regulation (hsa04923);

Signaling: Includes endocannabinoid signaling (hsa04723) and cytoskeletal regulation (hsa04820).

2. " - " indicates not applicable/no pathway association.

These findings provide further evidence, at the pathological association level, for the divergent disease association profiles of the metabolic-immune adaptation system versus the circadian rhythm regulatory system in the pathology of sustained hypoxia-induced heart disease. Even when the FDR criterion was relaxed, the former establishes broad associations with cardiac diseases through multi-pathway, multi-gene cooperative regulatory networks, whereas the latter's association with cardiac diseases is manifested solely through signaling by a single adenosine receptor gene (ADORA2A), and this association fails to achieve statistical significance under conventional algorithmic thresholds.

### 3.6 Cardiovascular drug target analysis: divergent therapeutic profiles of the two systems

To further evaluate the potential therapeutic value of targeting these two distinct response systems, we performed systematic drug enrichment analysis by integrating four drug-gene target interaction databases. Initial enrichment screening identified 61 drugs significantly associated with the core genes derived from the whole-transcriptome GSEA of the sustained hypoxic right ventricle, and 11 drugs significantly associated with the core genes corresponding to the eight circadian differentially expressed genes, both at an FDR threshold of 0.05. Following cardiovascular-related keyword matching and manual curation (removing non-cardiovascular drugs such as dronabinol, and retaining experimental compounds with caution), 60 drugs from the GSEA-derived set and 11 drugs from the circadian gene-derived set were identified as relevant to cardiovascular therapy.

Examination of the drug type distribution revealed distinct pharmacological profiles between the two systems. The 60 GSEA-enriched cardiovascular drugs spanned eleven major categories: lipid-lowering agents, calcium channel blockers, beta-blockers, alpha-blockers, cardiotonics, vasoactive agents/vasopressors, antiarrhythmics, antiplatelet/anticoagulant, diuretics, vasodilators, and metabolic modulators (Table 2). In contrast, the 11 circadian gene-enriched cardiovascular drugs comprised six categories: calcium channel blockers, alpha-blockers, vasoactive agents/vasopressors, antiarrhythmics, diuretics, and vasodilators. Cross-analysis between the two drug sets revealed only 6 overlapping drugs (Table 2), which included calcium channel blockers such as nifedipine and nitrendipine; alpha-blockers including indoramin and fenoldopam; and the antiarrhythmic drugs amiodarone and adenosine. From a pathway perspective, among the KEGG pathways associated with these 6 overlapping drugs from the GSEA-derived set, the cardiovascular-related pathways included hsa04261 (Adrenergic signaling in cardiomyocytes), hsa04260 (Cardiac muscle contraction), hsa05412 (Arrhythmogenic right ventricular cardiomyopathy), hsa04082 (Neuroactive ligand signaling), hsa05415 (Diabetic cardiomyopathy), hsa04146 (Peroxisome), and hsa01212 (Fatty acid metabolism). The circadian gene-derived drugs, however, failed to enrich to any specific pathways due to the limited number of input genes.

**Table 2:** Summary of enriched drugs related to cardiovascular diseases.

Category	Enrichment from GSEA Pathways (n = 72)	Enrichment from hypoxia-responsive circadian genes (n = 12)
Lipid-Lowering Agents	Simvastatin, Lovastatin, Atorvastatin, Pravastatin, Rosuvastatin	—
Calcium Channel Blockers	**Nifedipine** (hsa04260, hsa04082, hsa04261, hsa04723, hsa04923, hsa04930, hsa05020, hsa05410, hsa05412, hsa05414)	**Nifedipine**
	**Nicardipine (hsa00630,** hsa04064, hsa04082, hsa04260, hsa04261, hsa04657, hsa04668, hsa04723, hsa04923, hsa04930, hsa05020, hsa05133, hsa05144, hsa05323, hsa05410, hsa05412, hsa05414, hsa05418)	**Nicardipine**
	**Nitrendipine** (hsa04260, hsa04082, hsa04261, hsa04723, hsa04923, hsa04930, hsa05020, hsa05410, hsa05412, hsa05414)	**Nitrendipine**
	Nisoldipine, Nimodipine, Verapamil	—
Beta-Blockers	Carvedilol, Atenolol, Metoprolol, Nebivolol, Bisoprolol	—
Cardiotonics/Vasoactive Agents	**Isoprenaline** (hsa04923, hsa04082)	**Isoprenaline**
	Ouabain, Lanatoside, Digitoxin, Epinephrine, Norepinephrine, Isoproterenol, Dopamine	—
Antiarrhythmics	**Amiodarone** (hsa04261, hsa05414, hsa04923, hsa04082)	**Amiodarone**
	Lidocaine	—
Antiplatelet/Anticoagulant Drugs	Clopidogrel, Warfarin, Enoxaparin, Heparin	—
Diuretics	Furosemide	Amiloride
Vasodilators	Nitroglycerin, Minoxidil	Pentoxifylline
Antihypertensives	**Phenoxybenzamine** (hsa04261, hsa04082)	**Phenoxybenzamine**
	**Indoramin** (hsa04261, hsa04082)	**Indoramin**
	**Fenoldopam** (hsa04261, hsa04082)	**Fenoldopam**
	Phenylephrine	—
Others	Dobutamine, Levosimendan, Milrinone, etc.	Ketanserin

Note: Overlapping drugs and pathways are indicated in bold; the related KEGG pathways are only listed after the overlapping drugs. hsa00630 (Glyoxylate and dicarboxylate metabolism).

Notably, the overlap between the two systems was highly asymmetric, that is only 6 of the 60 GSEA-derived cardiovascular drugs (10.0%) were shared with the circadian gene set, whereas 6 of the 11 circadian gene-derived cardiovascular drugs (54.5%) overlapped with the GSEA set. This disproportionate pattern of intersection provides further evidence, from a drug target perspective, that the metabolic-immune adaptation system and the circadian rhythm regulatory system exhibit divergent pharmacological profiles, with limited overlap in their associated cardiovascular drug targets.

## 4. Discussion

Our comparison of cardiac transcriptomic responses across four DEG sets revealed minimal overlap, with no shared DEGs among the sustained hypoxia, prenatal fetal, and adult subacute groups, and only 13 DEGs shared between the sustained hypoxia and prenatal adult programmed heart groups. The sustained hypoxia group (181 DEGs) and prenatal adult programmed heart group (635 DEGs) exhibited the most substantial transcriptional changes, whereas the prenatal fetal group produced only 2 DEGs, likely reflecting a limited transcriptional response, though the small sample size (n = 3 per group) limits statistical power. These findings indicate that distinct developmental windows and hypoxia exposure durations fundamentally differentiate the heart's response mechanisms, and are consistent with the established association between chronic hypoxia and the heart disease [21]. Elucidating the molecular adaptation mechanisms of the heart under lifelong hypoxia exposure holds substantial reference value for therapeutic strategy development in affected patients.

It is important to note that the five analysis groups differed in multiple experimental variables beyond hypoxia duration, including tissue type (whole heart vs. right ventricle vs. left ventricle), hypoxia model (hypobaric chamber vs. normobaric hypoxia), oxygen level (~14.5% vs. 10%), and sequencing platform (Supplementary S2 Table). These differences preclude direct attribution of all observed transcriptomic divergence to hypoxia exposure paradigms alone. In particular, the distinct circadian DEG sets observed in the sustained hypoxia and prenatal adult programmed heart groups may partly reflect differences in cardiac region (right ventricle vs. whole heart) rather than solely the developmental timing of hypoxia exposure.

Differential expression analysis revealed that the sustained hypoxia group contained 8 circadian DEGs (*Ass1*, *Bmal1*, *Drd4*, *Npas2*, *Per3*, *Adora2a*, *Id1*, *Per2*), and the prenatal adult programmed heart group contained 7 circadian DEGs (*Klf10*, *Cyp7b1*, *Rorb*, *Scn9a*, *Kcnh7*, *Nlgn1*, *Slc6a4*), whereas neither the prenatal hypoxia embryonic hearts nor the adult subacute hypoxia hearts exhibited significant differential expression of circadian genes. The complete absence of overlap between the two circadian DEG sets suggests that the developmental timing of hypoxia exposure, rather than exposure per se, determines which circadian genes are affected, though differences in tissue type (whole heart vs. right ventricle) and age at sampling may also contribute to this divergence. Notably, the PH-Adult circadian DEGs were predominantly associated with neurotransmission and ion channel functions (e.g., Scn9a, Kcnh7, Slc6a4, Nlgn1), whereas the sustained hypoxia circadian DEGs comprised core clock components and metabolic regulators. This functional divergence further supports the notion that the biological consequences of circadian gene dysregulation depend on the developmental context of hypoxia exposure. Given that the circadian clock is known to maintain cardiac function through rhythmic coordination of metabolism, signal transduction, and electrophysiological processes [22], such coordination may be reprogrammed under chronic stress conditions. Furthermore, our analysis demonstrated that the 160 rhythm-associated interaction gene pairs identified under normoxic conditions dramatically decreased to only 52 pairs under hypoxia, representing a 67.5% reduction. This result suggests that sustained hypoxia may simplify the network structure by reducing redundant rhythm interactions, thereby achieving more efficient regulation.

The surviving core clock network within the sustained hypoxia group exhibited three categories of adaptive changes, as validated by permutation testing (n = 10,000). The first category, enhanced synchronicity, was exemplified by *Ass1*-*Mcemp1* (r: 0.437 to 0.983, Δr = +0.546, empirical p = 3.8 × 10^−3^) and *Ass1*-*Hpgd* (r: 0.178 to 0.982, Δr = +0.804, empirical p = 9.0 × 10^−4^), indicating strengthened metabolic coupling under hypoxia. The second category, interaction reversal, was exemplified by *Id1*-*Krt8* (r: 0.984 to 0.346, Δr = −0.639, empirical p = 2.2 × 10^−2^) and *Adora2a*-*Map3k7cl* (r: 0.964 to 0.239, Δr = −0.725, empirical p = 8.0 × 10^−3^), suggesting fundamental reorganization of metabolic-circadian coupling. The third category, weakened synchronicity, involved significant attenuation of relationships including *Adora2a*-*Lrrc9* (r: 0.897 to −0.177, Δr = −1.074, empirical p = 4.0 × 10^−3^) under hypoxia. Of note, the median absolute correlation coefficient in the hypoxia group reached 0.975, with 88.5% of interactions concentrated in the |r| > 0.97 interval, indicating a shift toward higher correlation coefficients under hypoxia. These changes may lead to alterations in clock output signaling.

Beyond these interaction pattern changes, a pronounced reorganization of hub genes within the circadian network was also observed. Under normoxic conditions, a dual-hub architecture predominated, with *Id1* (55 interaction targets) and *Adora2a* (81 interaction targets) emerging as the two most prominent hub genes, while the metabolism-associated gene *Ass1* accounted for only 11.25% of total interaction pairs, despite being downregulated in the sustained hypoxia group (log_2_FC = −1.44, FDR = 2.8 × 10^−3^). Remarkably, in the hypoxic environment, *Id1* (log_2_FC = −1.29, FDR = 3.5 × 10^−3^) and *Adora2a* (log_2_FC = −1.10, FDR = 4.5 × 10^−4^) completely vanished from the interaction network, while *Ass1’*s share of total interaction pairs surged to 88.5%, establishing it as the predominant hub gene in the co-expression network. Notably, this dramatic shift in topological centrality occurred independently of its expression level, as network hub status reflects connectivity patterns rather than transcript abundance. *Ass1* (argininosuccinate synthase 1) serves as a key enzyme in the urea cycle and is also a reported target gene of HIF1α. Studies by Silberman *et al.* (2019) demonstrated that ASS1 is downregulated by HIF1α under hypoxic conditions in tumor cells [23]. However, it is well-documented that HIF1α target genes exhibit pronounced tissue-specific and context-dependent patterns [24], and our finding reveals that under sustained cardiac hypoxia, *Ass1* instead emerges as a transcript-level co-expression hub within the circadian interaction network, suggesting that arginine metabolism may play a central role in cardiac hypoxia adaptation through coupling with the circadian system. This discovery echoes the study by Xu *et al.* (2025) regarding arginase 2 (Arg2) regulation of cardiovascular adaptation under hypoxia [25], indicating that arginine metabolic pathways hold important functions in hypoxic heart pathology. Nevertheless, the core oscillator components *Bmal1* and *Npas2* maintained a stable positive correlation under hypoxia (r = 0.838 vs. 0.829, Δr = −0.009), demonstrating that the core clock network retains considerable hypoxic resilience despite overall restructuring. This finding aligns with the report by Lecacheur *et al.* (2024) concerning the human heart clock [26]. Their review noted that while peripheral clock outputs (such as rhythms in metabolism and signaling pathway-related gene expression) undergo significant reprogramming under pathological conditions like aging or obesity, the expression rhythms of core oscillator components (such as BMAL1 and PER2) tend to remain relatively stable in the human heart. Moreover, the integrity of this core clock is crucial for maintaining basic cardiac function, as genetic studies have shown that disruption of the core clock directly leads to severe cardiac dysfunction.

Importantly, the metabolic-immune adaptation system and the circadian rhythm regulatory system exhibited divergent response patterns under sustained hypoxia. GSEA analysis identified 55 significantly enriched KEGG pathways (Supplementary S1 Table), with energy metabolism pathways (*e.g.*, oxidative phosphorylation, TCA cycle) showing positive enrichment and immune-inflammatory pathways (*e.g.*, IL-17 signaling, NF-κB signaling, TNF signaling) showing negative enrichment. This bidirectional pattern is consistent with cardiac hypoxia-induced metabolic reprogramming, where energy production is prioritized while immune responses are suppressed [27]. In contrast, the 8 circadian DEGs enriched only 3 pathways (circadian rhythm and neuroactive ligand-receptor interaction), with no overlap between the two enrichment tiers, suggesting distinct enrichment profiles at the pathway level, though we note that the absence of KEGG pathway overlap does not preclude functional interactions at other regulatory levels.

Beyond pathway enrichment differences, gene interaction strength also underwent systematic restructuring under hypoxic stress. Analysis revealed that the median absolute correlation coefficient for interaction pairs in the hypoxia group reached 0.975, representing a 5.2% increase compared to the normoxia group (median = 0.927, p < 0.001). Furthermore, the hypoxia group displayed a distinct “high-peaked” distribution, with interaction strength concentrated in the |r| > 0.97 interval (accounting for 88.5%), completely eliminating weak associations below |r| = 0.8. In contrast, the normoxia group retained a broader distribution of weaker associations. This shift toward higher correlation coefficients may reflect the streamlining and optimization of network regulation under hypoxic stress. However, Spearman rank correlation analysis yielded substantially lower coefficients than Pearson correlations (median |ρ| = 0.357 vs. median |r| = 0.976 in hypoxia; Supplementary S3 Table), and leave-one-out sensitivity analysis indicated that several Ass1-centric correlations were sensitive to single-sample removal. These findings suggest that the high Pearson correlations should be interpreted with appropriate caution, as they may be partly influenced by distributional properties or influential observations.

To further characterize the divergent profiles of these two systems, our study conducted analyses at both the disease and drug target levels. Based on disease enrichment analysis, there was absolutely no overlap in cardiac-related diseases between the 123 cardiac diseases identified from the whole-transcriptome GSEA of sustained hypoxia and the 2 cardiac diseases associated with circadian genes. Even when the FDR threshold was relaxed to 0.1, although 3 common diseases emerged (chronic heart failure, secondary myocardial diseases, and primary cardiomyopathies), detailed analysis revealed fundamental differences: the former involved 13–17 KEGG pathways exhibiting typical multi-pathway, polygenic regulatory network characteristics, while the latter represented single-gene enrichments (ADORA2A) with FDR values exceeding 0.05 and thus failing to meet conventional significance thresholds. Notably, although ADORA2A (adenosine A2A receptor) has been confirmed to participate in cardioprotective signaling [28], our study suggests it may serve as a “bystander” rather than a “driver” in the association between the circadian system and heart disease. In contrast, the metabolic-immune adaptation system establishes a closer association with heart disease through synergistic regulation of multiple pathways including energy metabolism, inflammatory signaling, and myocardial contraction.

Drug enrichment analysis further substantiated these conclusions. Only 6 of 60 GSEA-derived cardiovascular drugs (10.0%) overlapped with the circadian gene-derived set, whereas 6 of 11 circadian gene-derived drugs (54.5%) overlapped with the GSEA set, providing further evidence for the divergent pharmacological profiles of these two systems. These findings suggest that pharmacological intervention targeting the metabolic-immune system may simultaneously address circadian-related cardiac dysfunction, while the circadian system may have limited value as a standalone therapeutic target under sustained hypoxia. Although circadian rhythm plays an important role in cardiovascular health [6,29], our results suggest that under chronic sustained hypoxia, the metabolic-immune system may occupy a more dominant position, with the circadian system functioning more in an adjusting rather than driving role.

Given that the datasets included in this study lacked sampling time information, circadian gene analysis may have been confounded by this factor. To address this limitation, we employed permutation testing (n = 10,000) to assess whether the observed differences could be attributed to random sampling variation. The results demonstrated that core circadian gene expression changes were robust against random label permutation, with empirical p values consistently below 0.05 (Fig 4). At the network level, 85 of 211 gene pairs (40.3%) showed significantly altered correlations after permutation validation, with high-fidelity interactions including *Ass1*-*Hpgd* (r = 0.982, empirical p = 9.0 × 10^−4^) and *Ass1*-*Mcemp1* (r = 0.983, empirical p = 3.8 × 10^−3^). However, we acknowledge that permutation testing evaluates robustness against random label assignment and cannot fully substitute for recorded sampling time information. If sample collection times differed systematically between hypoxia and control groups, the observed circadian gene expression changes could partially reflect circadian phase differences rather than hypoxia-specific effects. This limitation is inherent to all retrospective analyses of publicly available transcriptomic datasets for circadian biology and should be considered when interpreting the findings.

In summary, our study systematically revealed the transcriptomic response characteristics of rat hearts under lifelong sustained hypoxia exposure, identified *Ass1* as a transcript-level co-expression hub within the circadian interaction network under hypoxia, clarified the divergent profiles of the metabolic-immune adaptation system and the circadian rhythm regulatory system at the levels of pathways, diseases, and drug targets, and enhanced the reliability of our conclusions through statistical control of sampling time confounding factors. These findings provide novel insights into understanding cardiac molecular adaptation mechanisms under chronic hypoxia and offer potentially valuable intervention target references for therapeutic strategies in affected patients.

Nevertheless, certain limitations of this study warrant acknowledgment. First, our analysis was based on transcriptomic data from public databases, lacking validation at the protein expression and functional experimental levels. Second, the study subjects were rat models, and the translational relevance of these findings to human cardiac pathology requires further validation. Third, sampling time information was not recorded in the original studies, and while permutation testing was used to assess robustness against random sampling variation, it cannot fully exclude systematic sampling time differences between groups. Fourth, the prenatal adult programmed heart group (GSE129848) was analyzed for differential expression but was not included in the co-expression network analysis due to its distinct biological paradigm (prenatal programming vs. lifelong exposure) and tissue type (whole heart vs. right ventricle). The 7 circadian DEGs identified in this group, which are entirely non-overlapping with those from the sustained hypoxia group, warrant dedicated investigation in future studies. More broadly, the absence of independent validation datasets with both chronic hypoxia exposure and recorded circadian sampling time information limits the generalizability of the observed circadian network remodeling. Fifth, the co-expression network was inferred from a modest sample size (n = 13 hypoxia, n = 11 normoxia), and Spearman rank correlation analysis yielded substantially lower coefficients than Pearson correlations (Supplementary S3 Table), suggesting that the network topology should be interpreted with appropriate caution. Future research could further validate the core findings at the protein level and explore the translational significance of the Ass1-circadian interaction network in clinical samples.

## Supporting information

S1 Table Pathways identified in sustained hypoxic right ventricle through GSEA analysis (n = 55). Positive NES indicates up-regulation under hypoxia; negative NES indicates down-regulation. All pathways listed at FDR < 0.05.
(DOCX)

S2 Table Overview of the five transcriptomic datasets analyzed in this study. Datasets were retrieved from NCBI GEO and organized into five analysis groups spanning three distinct hypoxia paradigms. Key experimental variables, including developmental stage, tissue type, hypoxia model, oxygen level, exposure duration, and age at sampling, differ substantially across datasets. The Prenatal_Adult_Heart group (GSE129848 adult offspring) represents prenatal hypoxia programming effects assessed at 5 months postnatal age under normoxic conditions.
(DOCX)

S3 Table Robustness validation of key circadian gene co-expression pairs under sustained hypoxia. Pearson and Spearman correlation coefficients, 95% confidence intervals (Fisher's z-transformation), and leave-one-out sensitivity ranges for key circadian gene pairs. Global comparison of Pearson and Spearman median |r| values is shown at the bottom.
(DOCX)
