# Supplementary material for: Sustained hypoxia induces divergent response patterns in cardiac metabolic-immune adaptation and circadian rhythm regulation
Source: PLoS One. 2026 Sep 11;21(9):e0357966. doi: 10.1371/journal.pone.0357966 (PMC13567775; doi:10.1371/journal.pone.0357966)
Supplement: Supplementary file 1 [file pone.0357966.s001.docx]

**Supplementary Table 1. Pathways identified in sustained hypoxic right ventricle through GSEA analysis (n = 55)**

| **ID** | **Description** | **setSize** | **enrichmentScore** | **NES** | **FDR** | **Genes** |
| --- | --- | --- | --- | --- | --- | --- |
| hsa00190 | Oxidative phosphorylation - Homo sapiens (human) | 89 | 0.57 | 2.45 | 3.76×10^-7^ | ATP4A/NDUFB8/ATP12A/CYC1/NDUFS1/COX6A2/SDHD/UQCRFS1/COX8A/NDUFB6/SDHA/NDUFA9/NDUFB11/COX5A/SDHC/ATP6V1D/NDUFB5/COX6B1/ATP6AP1/PPA1/COX4I1/UQCRC1/UQCRC2/NDUFS2/CYCS/NDUFAB1/NDUFC2/NDUFV1/NDUFA11/NDUFS3/NDUFB4/ATP6V0A2/COX5B/ATP6V0E2/SDHB/PPA2/NDUFA12/NDUFA10/NDUFB10/ATP6V1F/NDUFS8/NDUFA8/NDUFV3/COX6A1/NDUFS5/UQCR11/COX11/COX7C/ATP6V0C/UQCRH/ATP6V0B/NDUFV2/NDUFS6/ATP6V1E1/NDUFS4/COX7A2/UQCR10/NDUFA7/ATP6V1A/NDUFA4 |
| hsa01200 | Carbon metabolism - Homo sapiens (human) | 92 | 0.55 | 2.39 | 6.34×10^-7^ | ACAT2/SHMT1/FBP2/ENO3/PKM/ACSS1/MDH2/GPT/GAPDH/GOT2/PFKM/PGK1/IDH2/PCCA/MDH1/SDHD/ALDH6A1/SDHA/IDH3G/PDHB/OGDH/GPT2/ME3/IDH3A/ACADS/PC/GOT1/ME1/DLST/GLYCTK/MCEE/ALDOA/SDHC/CS/GPI/GCSH/ACSS2/PDHA1/SUCLG1/DLD/DLAT/H6PD/ACO2/ENO1/FH/SUCLG2/SDHB |
| hsa00620 | Pyruvate metabolism - Homo sapiens (human) | 36 | 0.71 | 2.54 | 8.25×10^-6^ | ACAT2/ADH7/LDHA/PKM/ACSS1/MDH2/ACACB/MDH1/PDHB/ACYP2/ME3/ALDH2/PC/ME1/LDHB/ACSS2/PCK1/PDHA1/DLD/DLAT/FH/LDHD/ALDH7A1/ALDH9A1 |
| hsa00020 | Citrate cycle (TCA cycle) - Homo sapiens (human) | 28 | 0.74 | 2.53 | 9.16×10^-6^ | MDH2/IDH2/MDH1/SDHD/SDHA/IDH3G/PDHB/OGDH/IDH3A/PC/DLST/SDHC/CS/PCK1/PDHA1/SUCLG1/DLD/DLAT/ACO2/FH/SUCLG2/SDHB/SUCLA2/ACO1 |
| hsa00280 | Valine, leucine and isoleucine degradation - Homo sapiens (human) | 39 | 0.66 | 2.43 | 9.16×10^-6^ | ACAT2/OXCT1/MCCC2/PCCA/ALDH6A1/HADHB/ALDH2/ACADS/BCAT2/HADH/MCEE/HADHA/ACAA2/BCKDHB/HSD17B10/IVD/DLD/MCCC1/HIBADH/ALDH7A1/ALDH9A1/EHHADH/PCCB/ACADM/AUH/DBT/BCAT1/HIBCH/ACAT1/BCKDHA/ACADSB |
| hsa05415 | Diabetic cardiomyopathy - Homo sapiens (human) | 159 | 0.43 | 2.06 | 9.16×10^-6^ | CAMK2A/SLC2A4/CYBB/GYS1/ATP2A2/NDUFB8/GAPDH/SLC2A1/PRKCA/CYC1/SLC25A5/VDAC1/NDUFS1/COX6A2/SDHD/UQCRFS1/CPT1B/COX8A/PLN/NCF1/INSR/NDUFB6/SDHA/PDHB/MPC1/PIK3R1/NDUFA9/NDUFB11/COX5A/SDHC/PPARA/NDUFB5/COX6B1/COX4I1/UQCRC1/UQCRC2/PDK2/NDUFS2/NDUFAB1/PDHA1/SLC25A4/NDUFC2/NDUFV1/AKT1/NDUFA11/NDUFS3/VDAC2/NDUFB4/GFPT1/PPIF/COX5B/PDK4/VDAC3/CTSD/CPT2/SDHB/NDUFA12/NDUFA10/NDUFB10/AKT2/NDUFS8/NDUFA8/NDUFV3/COX6A1/NDUFS5/UQCR11/COX7C/PLCB2/IRS1/UQCRH/NDUFV2/MPC2/NDUFS6 |
| hsa00640 | Propanoate metabolism - Homo sapiens (human) | 26 | 0.73 | 2.40 | 5.74×10^-5^ | LDHA/ACSS1/ACACB/PCCA/ALDH6A1/ACADS/LDHB/MCEE/ACSS2/HADHA/MLYCD/BCKDHB/SUCLG1/ACSS3/DLD/SUCLG2/SUCLA2/EHHADH/PCCB/ACACA/DBT/HIBCH/BCKDHA |
| hsa04820 | Cytoskeleton in muscle cells - Homo sapiens (human) | 170 | 0.39 | 1.91 | 1.14×10^-4^ | TNNI1/MYL7/MYBPHL/MYL4/MYBPC2/ACTC1/ACTA1/TCAP/SPTBN4/ATP1A1/CKM/ENO3/MYH7B/MYL2/CSRP3/TNNC1/MYH6/THBS1/MYPN/TMOD1/FLNC/ATP1B4/FHL1/MYL3/MYH14/ATP1B1/ACTN2/ITGB3/SYNPO2/SGCA/TPM1/NRAP/MYOM1/VCAN/PDLIM5/MYOZ2/DMD/SGCG/FHL2/ITGA4/DES/SGCB/MYBPC3/ITGA7/COL4A4/AMPD3/COL4A3/TRIM55/COL5A1/PDLIM7/DSC2/SYNE3/ENO1/PLEC/LMNB1/LDB3/COMP/SDC4/SNTA1/MYOM2/SSPN |
| hsa01210 | 2-Oxocarboxylic acid metabolism - Homo sapiens (human) | 26 | 0.70 | 2.31 | 2.09×10^-4^ | GPT/GOT2/IDH2/IDH3G/PDHB/OGDH/GPT2/IDH3A/GOT1/DLST/BCAT2/CS/BCKDHB/PDHA1/DLD/ACY1/DLAT/ACO2 |
| hsa00630 | Glyoxylate and dicarboxylate metabolism - Homo sapiens (human) | 24 | 0.71 | 2.29 | 2.23×10^-4^ | ACAT2/SHMT1/GLUL/ACSS1/MDH2/PCCA/MDH1/GLYCTK/MCEE/CS/GCSH/ACSS2/DLD/ACO2/AFMID/PCCB/ACO1 |
| hsa05171 | Coronavirus disease - COVID-19 - Homo sapiens (human) | 182 | -0.49 | -1.80 | 2.23×10^-4^ | RPL4/RPL21/RPS23/RPL34/RPL28/RPS3A/RPL36A/IL6ST/MAPK13/RPS25/RPL13/C1R/RPL26/RPL39/MAPK11/RPS8/PLCG1/RPL32/RPL6/RPS24/RPL11/RELA/RPS5/RPL30/RPL23A/IFNAR1/FCGR2A/RPS15A/RPL35/RPS14/RPL35A/CFD/OAS2/IRF9/RPL22/PRKCG/CXCL10/RPL13A/FAU/IFIH1/RPS6/RPL38/C2/MAPK3/RPL31/RPL3/MAPK10/PIK3CA/CCL2/MYD88/TNFRSF1A/RPS12/IRAK4/VWF/RPS28/HBEGF/ADAM17/NFKBIA/C8G/ACE/JUN/CASP1/RPLP2/F2/RPS4Y2/OAS1/MX1/RPS19/IL1B/ISG15/C9/CFB/SELP/FOS/FGG/TMPRSS2/FGB/FGA |
| hsa04714 | Thermogenesis - Homo sapiens (human) | 167 | 0.38 | 1.83 | 2.97×10^-4^ | PLIN1/NPPA/NDUFB8/CREB3L2/CYC1/NDUFS1/COX6A2/SDHD/ADCY5/UQCRFS1/CPT1B/COX8A/NDUFB6/SDHA/NDUFAF7/NDUFA9/NDUFB11/COX5A/SDHC/NDUFB5/COX6B1/PRKG1/COX4I1/UQCRC1/UQCRC2/ACSL6/NDUFS2/ADCY7/PRKAB2/NDUFAB1/NDUFC2/NDUFV1/NDUFA11/TSC1/NDUFS3/NDUFB4/COX5B/ACSL1/SLC25A20/ADCY2/CPT2/SDHB/NDUFA12/PRKAB1/NDUFA10/SMARCD3/PRKACA/NDUFB10/COA5/SLC25A29/RPTOR/NDUFS8/NDUFA8/NDUFV3/COX6A1/NDUFS5/UQCR11/COX11/NDUFAF3/COX7C/NDUFAF1/UQCRH/NDUFV2/NDUFS6/CPT1C/MLST8/PRKAG2/PRKAA2/NDUFS4/COX7A2/NDUFAF6/ADCY1/CPT1A/UQCR10/NDUFAF4/CREB1/NDUFA7/NDUFA4 |
| hsa03272 | Virion - Hepatitis viruses - Homo sapiens (human) | 29 | -0.73 | -2.03 | 5.01×10^-4^ | DPP4/SCARB1/CLDN15/CD9/OCLN/CLDN5/CLDN22/CLDN18/EPCAM/CLDN3 |
| hsa00071 | Fatty acid degradation - Homo sapiens (human) | 35 | 0.62 | 2.21 | 5.53×10^-4^ | ACAT2/ADH7/CPT1B/HADHB/ALDH2/ACADS/HADH/HADHA/ACSL6/ACAA2/ECI2/ACSL1/CPT2/ALDH7A1/ACADVL/ALDH9A1/EHHADH/ACADM/CPT1C/ACADL/ECI1/CPT1A/ACAT1/ACADSB/ADH5 |
| hsa04260 | Cardiac muscle contraction - Homo sapiens (human) | 67 | 0.50 | 2.04 | 5.53×10^-4^ | CACNG6/MYL4/ACTC1/ATP1A1/MYL2/TNNC1/MYH6/ATP2A2/ATP1B4/SLC8A1/MYL3/ATP1B1/CYC1/TPM1/COX6A2/UQCRFS1/COX8A/CACNA1D/CASQ2/SLC9A7/COX5A/COX6B1/CACNB2/COX4I1/UQCRC1/UQCRC2 |
| hsa04610 | Complement and coagulation cascades - Homo sapiens (human) | 57 | -0.62 | -1.95 | 6.04×10^-4^ | THBD/VWF/C8G/PROCR/VTN/SERPINC1/F2/PLAT/C9/CFB/FGG/C4BPA/F10/SERPINA1/FGB/F9/FGA |
| hsa04723 | Retrograde endocannabinoid signaling - Homo sapiens (human) | 104 | 0.43 | 1.93 | 6.04×10^-4^ | RIMS1/GNG7/GRIA4/SLC17A7/GNAO1/GABRA3/NDUFB8/PRKCA/KCNJ5/NDUFS1/ADCY5/CACNA1D/NDUFB6/GNG3/NDUFA9/NDUFB11/DAGLA/NDUFB5/GRIA1/NDUFS2/PTGS2/ADCY7/NDUFAB1/NDUFC2/NDUFV1/NDUFA11/GRIA3/NDUFS3/NDUFB4/NAPEPLD/CACNA1A/GNB3/ADCY2/CACNA1C/NDUFA12/NDUFA10/PRKACA/NDUFB10/NDUFS8/NDUFA8/NDUFV3/NDUFS5 |
| hsa04060 | Cytokine-cytokine receptor interaction - Homo sapiens (human) | 152 | -0.50 | -1.79 | 7.14×10^-4^ | CCL1/CXCL12/TGFB2/BMP3/CXCL10/ACVR2B/IL1R2/LTBR/IL27RA/IL18/CCL3/CD40/CCL7/ACKR3/XCL1/CCL2/CCL4/TNFRSF1A/BMP6/IL15/IL33/EBI3/IL3RA/FAS/INHBB/ACVRL1/CXCL16/TNFSF12/BMP15/BMP4/IL2RG/CCR5/IL34/RELT/TGFB1/TNFRSF11A/IL17RE/TNFSF10/PF4/GDF10/IL16/XCR1/CCL22/CCL24/NGF/TNFRSF9/CSF2RB/IL1B/CCL11/CCL13/CXCR4/CXCL13/CCL19/CCL21/CXCL1/CXCL17/CXCL14 |
| hsa04382 | Cornified envelope formation - Homo sapiens (human) | 87 | -0.57 | -1.90 | 7.56×10^-4^ | ELOVL1/S100A10/ITGB4/COL4A1/KRT72/S100A11/ITGA6/KRT4/S100A16/S100A13/CLDN15/KRT77/ST14/ALOXE3/KRT80/OCLN/EVPL/S100A8/EPHX3/S100A9/KRT1/S100A3/KRT28/KRT19/CLDN5/KRT12/CLDN22/CLDN18/PRSS8/KRT8/KRT18/CLDN3 |
| hsa04979 | Cholesterol metabolism - Homo sapiens (human) | 37 | -0.69 | -2.00 | 8.61×10^-4^ | SCARB1/MYLIP/ANGPTL4/PLTP/APOE/LRP2/APOA1/APOC1/APOB/CIDEB/APOH/APOC3 |
| hsa04061 | Viral protein interaction with cytokine and cytokine receptor - Homo sapiens (human) | 56 | -0.61 | -1.91 | 1.55×10^-3^ | CCL1/CXCL12/CXCL10/LTBR/IL18/CCL3/CCL7/ACKR3/XCL1/CCL2/CCL4/TNFRSF1A/IL2RG/CCR5/IL34/TNFSF10/PF4/XCR1/CCL22/CCL24/CCL11/CCL13/CXCR4/CXCL13/CCL19/CCL21/CXCL1/CXCL14 |
| hsa01240 | Biosynthesis of cofactors - Homo sapiens (human) | 112 | 0.41 | 1.87 | 1.55×10^-3^ | UGT1A6/SHMT1/NMNAT2/MTHFD1L/PDXP/MTHFD2L/ALPL/NME4/PHOSPHO2/UROS/ALDH2/MTHFD1/BCAT2/PANK1/PNPO/UGP2/RDH13/AK1/MAT2A/LIAS/DLD/FLAD1/EARS2/COQ2/HMBS/PMM1/AK4/AFMID/CTPS1/GCLM/AK2/COQ7/DHODH/PMM2/COASY/COQ6/LIPT1/PDXK/ADSL/NME2/GMPPB/COQ3/MPI/PANK3/MTHFD2/UROD/BCAT1/PSAT1/NME6/ALAS1/ALAD/SHMT2/MAT1A |
| hsa04657 | IL-17 signaling pathway - Homo sapiens (human) | 58 | -0.59 | -1.86 | 2.54×10^-3^ | MAPK3/MAPK10/CCL7/CCL2/CASP8/TRADD/NFKBIA/LCN2/CASP3/FOSB/TRAF3IP2/IL17RE/JUN/S100A8/S100A9/IL1B/CCL11/HSP90AA1/DEFB4A/FOS/CXCL1 |
| hsa05016 | Huntington disease - Homo sapiens (human) | 221 | 0.31 | 1.54 | 3.16×10^-3^ | GRIA4/GPX1/DNAH1/TUBA4A/NDUFB8/KIF5C/CREB3L2/CYC1/SLC25A5/VDAC1/NDUFS1/COX6A2/SDHD/UQCRFS1/TUBB3/COX8A/NDUFB6/SDHA/TUBA8/NDUFA9/NDUFB11/COX5A/SDHC/NDUFB5/COX6B1/GRIA1/COX4I1/TUBB4B/UQCRC1/UQCRC2/NDUFS2/CYCS/NDUFAB1/SLC25A4/NDUFC2/SOD2/NDUFV1/MAP2K7/ERN1/NDUFA11/GRIA3/NDUFS3/VDAC2/NDUFB4/PPIF/COX5B/DLG4/TUBA1B/VDAC3/SDHB/PSMD14/NDUFA12/NDUFA10/NDUFB10/PSMB7/NDUFS8/NDUFA8/NDUFV3/COX6A1/CLTB/NDUFS5/UQCR11/COX7C/PLCB2/ACTR1B/PSMC2/UQCRH/ATG13/NDUFV2/MAP3K10/NDUFS6/WIPI1/PSMD3/PSMD6/PSMD12/DNAL1/NDUFS4/ITPR1/COX7A2/UQCR10/ULK1/PSMB5/CREB1/NDUFA7/TFAM/NDUFA4/PSMA1 |
| hsa05020 | Prion disease - Homo sapiens (human) | 202 | 0.33 | 1.61 | 5.34×10^-3^ | HSPA1A/TUBA4A/CYBB/NDUFB8/KIF5C/CREB3L2/CYC1/SLC25A5/VDAC1/NDUFS1/COX6A2/SDHD/UQCRFS1/TUBB3/COX8A/CACNA1D/NCF1/CAV3/NDUFB6/SDHA/TUBA8/PIK3R1/NDUFA9/HSPA5/NDUFB11/COX5A/SDHC/NDUFB5/COX6B1/STIP1/COX4I1/TUBB4B/UQCRC1/UQCRC2/NDUFS2/CYCS/NDUFAB1/SLC25A4/NDUFC2/NCAM1/NDUFV1/NDUFA11/NDUFS3/VDAC2/NDUFB4/PPIF/COX5B/TUBA1B/VDAC3/SDHB/PSMD14/CACNA1C/NDUFA12/NDUFA10/PRKACA/NDUFB10/HSPA8/PSMB7/NDUFS8/NDUFA8/NDUFV3/COX6A1/NDUFS5/UQCR11/IL1A/COX7C/PSMC2/UQCRH/NDUFV2/NDUFS6/CSNK2B/PSMD3/PSMD6/PSMD12/GRIN2D/PIK3R3/C6/NDUFS4/ITPR1/COX7A2/UQCR10/PSMB5/CREB1/C1QB/NDUFA7/NDUFA4/PSMA1/CSNK2A1/NCAM2/PPP3R1/PSMA5 |
| hsa00785 | Lipoic acid metabolism - Homo sapiens (human) | 16 | 0.72 | 2.06 | 5.41×10^-3^ | PDHB/OGDH/DLST/GCSH/BCKDHB/PDHA1/LIAS/DLD/DLAT/LIPT1/AMT/DBT/BCKDHA/OGDHL/DHTKD1 |
| hsa04514 | Cell adhesion molecule (CAM) interaction - Homo sapiens (human) | 105 | -0.51 | -1.76 | 5.74×10^-3^ | NEGR1/CLDN1/ALCAM/VSIR/HLA-DQA1/NRCAM/SPN/MPZ/SLITRK1/CD99/HLA-DRA/CD6/CD40/ITGA6/HLA-DOB/HLA-DRB1/CDH5/CD226/VCAM1/CLDN15/F11R/NRXN1/NRXN2/SLITRK5/HLA-F/JAM2/OCLN/LRRC4/PDCD1LG2/ICAM2/PECAM1/ICAM1/ESAM/NTNG1/HLA-E/CLDN5/CDH1/SELP/MADCAM1/CLDN22/CLDN18/SDC1/CLDN3 |
| hsa04350 | TGF-beta signaling pathway - Homo sapiens (human) | 82 | -0.53 | -1.75 | 6.82×10^-3^ | TGFB2/HFE/TGIF1/PPP2R1B/NCOR1/ACVR2B/SMAD6/HAMP/MAPK3/CREBBP/BMP6/ID2/HDAC1/INHBB/BMP4/SMAD7/NRROS/TGFB1/LTBP1/TGIF2/ROCK1/ID4/SMAD1/LRRC32/FMOD/FST/ID3/ID1/GREM2/CDKN2B |
| hsa05160 | Hepatitis C - Homo sapiens (human) | 113 | -0.49 | -1.71 | 6.87×10^-3^ | YWHAQ/RELA/TP53/IFNAR1/PPP2R2B/OAS2/IRF9/CLDN1/CXCL10/PPP2R1B/MAPK3/PIK3CA/RIPK1/KRAS/TNFRSF1A/SOCS3/BAX/FAS/NR1H3/CASP8/TRADD/SCARB1/NFKBIA/CLDN15/CASP3/OCLN/CDKN1A/IRF7/OAS1/MX1/RSAD2/CLDN5/CLDN22/CLDN18/CLDN3/IFIT1B |
| hsa05414 | Dilated cardiomyopathy - Homo sapiens (human) | 81 | 0.42 | 1.78 | 6.92×10^-3^ | CACNG6/ADRB1/ACTC1/MYL2/TNNC1/MYH6/ATP2A2/SLC8A1/MYL3/ITGB3/SGCA/TPM1/ADCY5/PLN/CACNA1D/CAV3/DMD/SGCG/NOS1/ITGA4/DES/SGCB/MYBPC3/ITGA7/CACNB2/ADCY7/ADCY2/SNTA1/CACNA1C/SSPN/PRKACA |
| hsa05202 | Transcriptional misregulation in cancer - Homo sapiens (human) | 135 | -0.47 | -1.68 | 7.46×10^-3^ | CDKN1B/DUSP6/RARA/KLF3/NCOR1/IL1R2/POLK/NFKBIZ/CDKN2C/BCL6/CEBPA/CD40/ZEB1/BCL2A1/ID2/TCF3/RUNX1T1/BAX/HDAC1/SLC45A3/TRAF1/BIRC3/FLI1/ERG/FLT3/FLT1/SMAD1/H3-3B/IGFBP3/LMO2/IGF1/TSPAN7/CDKN1A/MYCN/PPARG/PLAT/PROM1/WT1/TLX1/HHEX/LYL1/SPINT1/HPGD/PAX5/TMPRSS2 |
| hsa00062 | Fatty acid elongation - Homo sapiens (human) | 22 | 0.64 | 1.99 | 9.79×10^-3^ | HACD1/ELOVL2/TECR/HADHB/HADH/PPT2/THEM4/ACOT2/HSD17B12/HADHA/ACAA2/ELOVL4 |
| hsa05418 | Fluid shear stress and atherosclerosis - Homo sapiens (human) | 119 | -0.47 | -1.64 | 1.01×10^-2^ | PRKCZ/ACVR2B/IL1R2/ARHGEF2/CALM1/MAPK10/PIK3CA/CCL2/TRPV4/TNFRSF1A/PDGFB/THBD/CTSL/BMP4/CAV1/CDH5/VCAM1/KDR/GSTP1/NOS3/GSTT1/JUN/CYBA/CAV2/DUSP1/EDN1/PECAM1/ICAM1/PLAT/IL1B/MGST2/HSP90AA1/KLF2/CALML3/FOS/ASS1/SDC1 |
| hsa05150 | Staphylococcus aureus infection - Homo sapiens (human) | 41 | -0.60 | -1.79 | 1.15×10^-2^ | HLA-DQA1/HLA-DRA/C2/HLA-DOB/HLA-DRB1/FCGR3A/ICAM1/FCGR2B/KRT28/KRT19/CFB/DEFB4A/KRT12/SELP/FGG/KRT18 |
| hsa04923 | Regulation of lipolysis in adipocytes - Homo sapiens (human) | 43 | 0.49 | 1.81 | 1.34×10^-2^ | PLIN1/CIDEC/NPPA/ADRB1/PTGER3/ADCY5/IRS2/INSR/PIK3R1/PRKG1/ADORA1/PTGS2/ADCY7/AKT1/ADCY2/PRKACA/AKT2 |
| hsa04261 | Adrenergic signaling in cardiomyocytes - Homo sapiens (human) | 128 | 0.36 | 1.68 | 1.42×10^-2^ | CACNG6/MYL4/ADRB1/ACTC1/ADRA1A/SCN5A/ATP1A1/KCNQ1/CAMK2A/MYL2/TNNC1/MYH6/ATP2A2/PIK3R5/ATP1B4/SLC8A1/CREB3L2/MYL3/ATP1B1/PRKCA/TPM1/ADRA1B/ADCY5/PLN/CACNA1D/CACNB2/ADCY7/SCN1B/AKT1/BVES/ADCY2/ADRA1D/BCL2/CACNA1C/PRKACA/AKT2 |
| hsa05010 | Alzheimer disease - Homo sapiens (human) | 291 | 0.28 | 1.44 | 1.66×10^-2^ | SLC39A2/TUBA4A/CYBB/ATP2A2/NDUFB8/GAPDH/KIF5C/WNT9B/CYC1/SLC25A5/VDAC1/NDUFS1/COX6A2/SDHD/UQCRFS1/TUBB3/COX8A/IRS2/CACNA1D/INSR/NDUFB6/SDHA/TUBA8/NOS1/PIK3R1/NDUFA9/NDUFB11/COX5A/SDHC/NDUFB5/COX6B1/DVL3/ATF6/FZD7/COX4I1/TUBB4B/UQCRC1/UQCRC2/IKBKG/NDUFS2/PTGS2/CYCS/NDUFAB1/SLC25A4/IDE/NDUFC2/NDUFV1/MAP2K7/ERN1/HSD17B10/WNT2B/AKT1/NDUFA11/LPL/NDUFS3/VDAC2/NDUFB4/PPIF/COX5B/TUBA1B/VDAC3/FZD3/LRP6/SDHB/PSMD14/CACNA1C/NDUFA12/NDUFA10/SLC11A2/WNT5A/NDUFB10/AKT2/ARAF/BACE1/PSMB7/NDUFS8/NDUFA8/NDUFV3/COX6A1/NDUFS5/UQCR11/IL1A/COX7C/PLCB2/FZD5/APBB1/IRS1/PSMC2/UQCRH/ATG13/NDUFV2/NDUFS6/WIPI1/SLC39A14/MAP2K2/CASP7/CSNK2B/PSMD3/PSMD6/PSMD12/SLC39A9/GRIN2D/PIK3R3/FZD1/NDUFS4/ATP2A1/ITPR1/COX7A2/UQCR10/CALM3/ULK1/XBP1/PSMB5 |
| hsa05412 | Arrhythmogenic right ventricular cardiomyopathy - Homo sapiens (human) | 62 | 0.42 | 1.70 | 1.87×10^-2^ | CACNG6/LEF1/ATP2A2/SLC8A1/ACTN2/ITGB3/SGCA/CACNA1D/CAV3/DMD/SGCG/NOS1/ITGA4/DES/SGCB/ITGA7/CACNB2/DSC2/SNTA1/CACNA1C/SSPN |
| hsa04146 | Peroxisome - Homo sapiens (human) | 73 | 0.41 | 1.70 | 1.91×10^-2^ | SLC27A2/PXMP4/DDO/GSTK1/IDH2/NUDT13/MLYCD/ACSL6/SOD2/ECI2/ACSL1/PRDX5/PECR/GNPAT/NUDT19/DHRS4/CRAT/PEX7/EPHX2/EHHADH/ECH1/NUDT12/PEX5/PXMP2/PHYH/HSD17B4/SCP2/CAT/PIPOX/SOD1/PEX2/PRDX1/ACOX1 |
| hsa00010 | Glycolysis / Gluconeogenesis - Homo sapiens (human) | 48 | 0.47 | 1.80 | 1.95×10^-2^ | ADH7/FBP2/LDHA/ENO3/PKM/ACSS1/GAPDH/PFKM/PGK1/PDHB/ALDH2/LDHB/ALDOA/PGM1/GPI/ACSS2/PCK1/PDHA1/DLD/DLAT/ENO1/ALDH7A1/G6PC3/ALDH9A1 |
| hsa01230 | Biosynthesis of amino acids - Homo sapiens (human) | 56 | 0.44 | 1.75 | 2.22×10^-2^ | PYCR1/SHMT1/GLUL/ENO3/PKM/GPT/CTH/GAPDH/GOT2/PFKM/PGK1/IDH2/IDH3G/GPT2/IDH3A/PC/GOT1/BCAT2/ALDOA/CS/MAT2A/ACY1/ACO2/ENO1 |
| hsa01212 | Fatty acid metabolism - Homo sapiens (human) | 48 | 0.46 | 1.77 | 2.35×10^-2^ | ACAT2/HACD1/ELOVL2/TECR/CPT1B/HADHB/ACADS/HADH/PPT2/HSD17B12/HADHA/ACSL6/ACAA2/ACSL1/ELOVL4/CPT2/ACADVL/EHHADH/ACADM/ACACA/CPT1C/ACADL/CPT1A/ACAT1/ACADSB/CBR4/FADS1/HSD17B4/SCP2/OXSM/MECR/MCAT/ACOX1 |
| hsa04064 | NF-kappa B signaling pathway - Homo sapiens (human) | 77 | -0.51 | -1.68 | 2.35×10^-2^ | ZAP70/CSNK2A2/PLCG1/LCK/LAT/RELA/BLNK/LYN/CXCL12/LTBR/CD40/RIPK1/CCL4/MYD88/TNFRSF1A/BCL2A1/BCL10/IRAK4/CARD10/TRADD/LBP/VCAM1/NFKBIA/TNFRSF11A/TRAF1/BIRC3/ICAM1/IL1B/CCL13/CCL19/CCL21/CXCL1 |
| hsa05208 | Chemical carcinogenesis - reactive oxygen species - Homo sapiens (human) | 172 | 0.31 | 1.48 | 2.36×10^-2^ | EPHX4/CBR1/NDUFB8/CYC1/SLC25A5/VDAC1/NDUFS1/COX6A2/SRC/SDHD/UQCRFS1/GSTA4/COX8A/GSTM2/NCF1/SLC26A2/NDUFB6/SDHA/PIK3R1/NDUFA9/NDUFB11/COX5A/SDHC/NDUFB5/COX6B1/COX4I1/UQCRC1/UQCRC2/IKBKG/NDUFS2/NDUFAB1/MGST3/SLC25A4/NDUFC2/SOD2/NDUFV1/MAP2K7/AKT1/NDUFA11/NDUFS3/VDAC2/NDUFB4/PPIF/COX5B/VDAC3/FOXO3/SDHB/NDUFA12/NDUFA10/NDUFB10/AKT2/ARAF/PTPN1/NDUFS8/PTPN11/EPHX2/NDUFA8/NDUFV3/COX6A1/HMOX1/NDUFS5/UQCR11/COX7C/UQCRH/NDUFV2/ABL1/NDUFS6/MAP2K2/ARNT |
| hsa04082 | Neuroactive ligand signaling - Homo sapiens (human) | 94 | 0.37 | 1.59 | 2.62×10^-2^ | DRD4/GNG7/ADRB1/GRIA4/SLC17A7/GNAO1/ADRA1A/GNAL/GABRA3/PRKCA/HTR1B/ADRA1B/ADCY5/P2RY1/PENK/GNG3/SLC17A9/GRIA1/CHRM2/ADORA1/ACHE/ADCY7/GRIA3/P2RY12/SLC1A7/GNB3/ADCY2/ADRA1D/PRKACA |
| hsa04670 | Leukocyte transendothelial migration - Homo sapiens (human) | 93 | -0.48 | -1.62 | 2.68×10^-2^ | MAPK12/CTNNB1/VASP/ACTG1/CLDN19/MAPK13/BCAR1/MAPK11/PLCG1/GNAI3/PTK2/JAM3/ARHGAP5/ACTB/RHOH/ITK/CLDN1/CXCL12/PRKCG/ACTN1/GNAI2/RAPGEF3/CD99/NCF4/PXN/PIK3CA/ACTN4/VAV3/CDH5/VCAM1/CLDN15/MYL12B/F11R/JAM2/ROCK1/AFDN/OCLN/CYBA/PECAM1/ICAM1/ESAM/CXCR4/CLDN5/CLDN22/CLDN18/CLDN3 |
| hsa04922 | Glucagon signaling pathway - Homo sapiens (human) | 86 | 0.37 | 1.59 | 2.81×10^-2^ | FBP2/LDHA/CAMK2A/GYS1/PKM/PYGM/CREB3L2/PFKM/ACACB/SLC2A1/GCGR/PYGB/CPT1B/PHKA2/PDHB/LDHB/PPARA/PCK1/PRKAB2/PDHA1/AKT1/PHKA1/ADCY2/PRKAB1/PRKACA/AKT2/PHKG1/G6PC3/PHKB/PLCB2/ACACA/PGAM1/CPT1C/PRKAG2/PRKAA2/ITPR1/CPT1A/CALM3/CREB1/CAMK2D/PPP3R1/PDE3B |
| hsa03030 | DNA replication - Homo sapiens (human) | 33 | 0.50 | 1.77 | 2.88×10^-2^ | MCM5/POLE/POLA2/MCM3/MCM2/POLE2/POLD2/MCM4/RPA1 |
| hsa04668 | TNF signaling pathway - Homo sapiens (human) | 99 | -0.48 | -1.64 | 2.88×10^-2^ | JUNB/MAPK3/CREB3L1/MAPK10/PIK3CA/RIPK1/CCL2/TNFRSF1A/SOCS3/IL15/FAS/IRF1/CASP8/TRADD/MMP14/VCAM1/ADAM17/NFKBIA/CASP3/TRAF1/BIRC3/BCL3/JUN/EDN1/VEGFC/JAG1/ICAM1/IL1B/FOS/CXCL1 |
| hsa00564 | Glycerophospholipid metabolism - Homo sapiens (human) | 77 | 0.38 | 1.63 | 2.88×10^-2^ | PLA2G2D/CDS1/PLA2G12A/DGKI/PHOSPHO1/PLA2G5/PCYT1B/SGMS2/SELENOI/PLA2G2C/PEMT/PLPP2/ACHE/PLB1/GNPAT/GPAM/GPD1L/PLA2G4B/LPIN1/PCYT2/LPGAT1/PCYT1A/GPD1/LPCAT3/SGMS1/CHPT1/AGPAT3 |
| hsa04550 | Signaling pathways regulating pluripotency of stem cells - Homo sapiens (human) | 95 | -0.46 | -1.58 | 3.49×10^-2^ | PCGF6/MAPK3/PIK3CA/KRAS/ID2/TCF3/TBX3/INHBB/FZD6/BMP4/SOX2/ID4/SMAD1/DLX5/IGF1/ID3/KLF4/FGFR2/ID1/WNT3A/NANOG |
| hsa00650 | Butanoate metabolism - Homo sapiens (human) | 16 | 0.65 | 1.85 | 3.50×10^-2^ | ACAT2/BDH1/OXCT1/ACADS/HADH/ALDH5A1/HADHA/L2HGDH |
| hsa05164 | Influenza A - Homo sapiens (human) | 120 | -0.44 | -1.55 | 3.50×10^-2^ | RELA/HLA-DOA/PABPN1/IFNAR1/ACTB/OAS2/IRF9/RAB11A/CXCL10/HLA-DQA1/PYCARD/IFIH1/IL18/HLA-DRA/MAPK3/CREBBP/PIK3CA/CCL2/MYD88/TNFRSF1A/SOCS3/IL33/BAX/FAS/IRAK4/HLA-DOB/HLA-DRB1/CASP8/TRADD/NFKBIA/CASP3/CASP1/TNFSF10/ICAM1/IRF7/OAS1/MX1/RSAD2/IL1B/TMPRSS2 |
| hsa05012 | Parkinson disease - Homo sapiens (human) | 210 | 0.29 | 1.41 | 3.89×10^-2^ | SLC39A2/TUBA4A/GNAL/CAMK2A/NDUFB8/KIF5C/CYC1/SLC25A5/VDAC1/NDUFS1/COX6A2/SDHD/ADCY5/UQCRFS1/TUBB3/COX8A/NDUFB6/SDHA/TUBA8/NDUFA9/HSPA5/NDUFB11/COX5A/SDHC/NDUFB5/COX6B1/ATF6/COX4I1/TUBB4B/UQCRC1/MFN2/UQCRC2/NDUFS2/CYCS/NDUFAB1/SLC25A4/NDUFC2/NDUFV1/ERN1/NDUFA11/NDUFS3/VDAC2/NDUFB4/PPIF/MAOA/COX5B/TUBA1B/VDAC3/SDHB/PSMD14/NDUFA12/NDUFA10/SLC11A2/TXN2/PRKACA/NDUFB10/PINK1/PSMB7/NDUFS8/NDUFA8/NDUFV3/COX6A1/TRAP1/NDUFS5/UQCR11/COX7C/PSMC2/UQCRH/NDUFV2/NDUFS6/SLC39A14/PSMD3/PSMD6/PSMD12/SLC39A9/NDUFS4/PRKN/ITPR1/COX7A2/PARK7/LRRK2/UQCR10/CALM3/XBP1/PSMB5/NDUFA7/NDUFA4/PSMA1/CAMK2D/UBA1 |
| hsa04932 | Non-alcoholic fatty liver disease - Homo sapiens (human) | 134 | 0.32 | 1.54 | 4.72×10^-2^ | NDUFB8/MLXIPL/CYC1/NDUFS1/COX6A2/SDHD/UQCRFS1/IL6R/COX8A/IRS2/INSR/NDUFB6/SDHA/PIK3R1/NDUFA9/NDUFB11/COX5A/SDHC/PPARA/NDUFB5/COX6B1/COX4I1/UQCRC1/UQCRC2/NDUFS2/PRKAB2/CYCS/NDUFAB1/NDUFC2/NDUFV1/ERN1/AKT1/NDUFA11/NDUFS3/NDUFB4/COX5B/SDHB/NDUFA12/PRKAB1/NDUFA10/NDUFB10/AKT2/RXRA/NDUFS8/NDUFA8/NDUFV3/COX6A1/NDUFS5/UQCR11/IL1A/COX7C/IRS1/UQCRH/NDUFV2/NDUFS6/CASP7/SREBF1/PRKAG2/PRKAA2/PIK3R3/NDUFS4/COX7A2/UQCR10/XBP1/ADIPOR2 |
