# Supplementary material for: Sustained hypoxia induces divergent response patterns in cardiac metabolic-immune adaptation and circadian rhythm regulation
Source: PLoS One. 2026 Sep 11;21(9):e0357966. doi: 10.1371/journal.pone.0357966 (PMC13567775; doi:10.1371/journal.pone.0357966)
Supplement: Supplementary file 2 [file pone.0357966.s002.docx]

| **Analysis_Group** | **GEO_ID** | **Tissue** | **Developmental_Stage** | **Hypoxia_Model** | **Hypoxia_Duration** | **O2_Level** | **Age_at_Sampling** | **Normoxia_n** | **Hypoxia_n** | **Platform** |
| --- | --- | --- | --- | --- | --- | --- | --- | --- | --- | --- |
| Sustained_Hypoxia_RV | GSE140146 | Right Ventricle | Adult (6 weeks) | Hypobaric chamber (~2743 m) | Sustained (fertilization → 6 weeks) | ~14.5% (hypobaric) | 6 weeks | 11 | 14 | HiSeq 4000 |
| Prenatal_Fetal_Heart | GSE129848 | Whole Heart | E21 (fetal) | Normobaric hypoxia (10% O₂) | Prenatal E15–E21 (6 days), then normoxia | 10% | E21 (0.6 weeks) | 3 | 3 | NextSeq 550 |
| Prenatal_Adult_Heart | GSE129848 | Whole Heart | Adult (5 months) | Normobaric hypoxia (10% O₂; prenatal only) | Prenatal E15–E21 (6 days), then normoxia  then normoxia | 10%→21% | 5 months (20–22 weeks) | 10 | 10 | NextSeq 550 |
| Subacute_Hypoxia_LV | GSE133402 | Left Ventricle | Adult (8–10 weeks) | Normobaric hypoxia (10% O₂) | Subacute (2 weeks) | 10% | 8–10 weeks | 3 | 3 | HiSeq 4000 |
| Subacute_Hypoxia_RV | GSE133402 | Right Ventricle | Adult (8–10 weeks) | Normobaric hypoxia (10% O₂) | Subacute (2 weeks) | 10% | 8–10 weeks | 3 | 3 | HiSeq 4000 |

**Supplementary Table S2. Overview of the five transcriptomic datasets analyzed in this study.**
