# Supplementary material for: Sustained hypoxia induces divergent response patterns in cardiac metabolic-immune adaptation and circadian rhythm regulation
Source: PLoS One. 2026 Sep 11;21(9):e0357966. doi: 10.1371/journal.pone.0357966 (PMC13567775; doi:10.1371/journal.pone.0357966)
Supplement: Supplementary file 3 [file pone.0357966.s003.docx]

**Supplementary Table S3. Robustness validation of key circadian gene co-expression pairs under sustained hypoxia.**

| **Gene Pair** | **Condition** | **n** | **Pearson r** | **95% CI (Pearson)** | **Spearman ρ** | **Leave-one-out \|r\| range** |
| --- | --- | --- | --- | --- | --- | --- |
| Arntl-Npas2 | Hypoxia | 13 | 0.833 | [0.522, 0.949] | 0.879 | 0.806-0.904 |
| Arntl-Npas2 | Normoxia | 11 | 0.855 | [0.525, 0.962] | 0.918 | 0.822-0.920 |
| Arntl-Per2 | Hypoxia | 13 | -0.802 | [-0.938, -0.451] | -0.819 | 0.734-0.870 |
| Arntl-Per2 | Normoxia | 11 | -0.390 | [-0.802, 0.274] | -0.591 | 0.026-0.754 |
| Per2-Per3 | Hypoxia | 13 | 0.780 | [0.403, 0.931] | 0.846 | 0.714-0.846 |
| Per2-Per3 | Normoxia | 11 | 0.406 | [-0.256, 0.809] | 0.555 | -0.090-0.902 |
| Per3-Ass1 | Hypoxia | 13 | -0.520 | [-0.833, 0.043] | -0.511 | 0.330-0.710 |
| Per3-Ass1 | Normoxia | 11 | -0.409 | [-0.810, 0.253] | -0.473 | 0.236-0.582 |
| Npas2-Ass1 | Hypoxia | 13 | 0.382 | [-0.214, 0.771] | 0.170 | 0.020-0.744 |
| Npas2-Ass1 | Normoxia | 11 | 0.500 | [-0.143, 0.846] | 0.600 | 0.379-0.621 |
| Adora2a-Id1 | Hypoxia | 13 | 0.148 | [-0.439, 0.646] | -0.055 | -0.041-0.337 |
| Adora2a-Id1 | Normoxia | 11 | 0.838 | [0.479, 0.957] | 0.418 | 0.720-0.956 |
| Ass1-Hpgd | Hypoxia | 13 | 0.982 | [0.940, 0.995] | 0.357 | 0.515-0.996 |
| Ass1-Hpgd | Normoxia | 11 | 0.768 | [0.312, 0.936] | 0.218 | 0.364-0.822 |
| Ass1-Mcemp1 | Hypoxia | 13 | 0.983 | [0.942, 0.995] | 0.632 | 0.584-0.997 |
| Ass1-Mcemp1 | Normoxia | 11 | 0.806 | [0.400, 0.948] | 0.591 | 0.511-0.872 |
| Ass1-Pigr | Hypoxia | 13 | 0.980 | [0.932, 0.994] | 0.527 | 0.820-0.998 |
| Ass1-Pigr | Normoxia | 11 | 0.782 | [0.343, 0.941] | 0.491 | 0.440-0.826 |

Note: Global comparison: In the hypoxia group (52 significant interaction pairs), the median Pearson |r| was 0.976 and the median Spearman |ρ| was 0.357. In the normoxia group (160 significant interaction pairs), the median Pearson |r| was 0.890 and the median Spearman |ρ| was 0.600.
